# *Mycobacterium tuberculosis*: immune response, biomarkers, and therapeutic intervention

**DOI:** 10.1002/mco2.419

**Published:** 2024-01-06

**Authors:** Li Zhuang, Ling Yang, Linsheng Li, Zhaoyang Ye, Wenping Gong

**Affiliations:** ^1^ Beijing Key Laboratory of New Techniques of Tuberculosis Diagnosis and Treatment Senior Department of Tuberculosis, the Eighth Medical Center of PLA General Hospital Beijing China; ^2^ Senior Department of Tuberculosis Hebei North University Zhangjiakou Hebei China

**Keywords:** biomarkers, diagnostic methods, immune checkpoints, immune response, mycobacterium tuberculosis (MTB), therapeutic vaccines, tuberculosis (TB)

## Abstract

Although tuberculosis (TB) is an infectious disease, the progression of the disease following *Mycobacterium tuberculosis* (MTB) infection is closely associated with the host's immune response. In this review, a comprehensive analysis of TB prevention, diagnosis, and treatment was conducted from an immunological perspective. First, we delved into the host's immune response mechanisms against MTB infection as well as the immune evasion mechanisms of the bacteria. Addressing the challenges currently faced in TB diagnosis and treatment, we also emphasized the importance of protein, genetic, and immunological biomarkers, aiming to provide new insights for early and personalized diagnosis and treatment of TB. Building upon this foundation, we further discussed intervention strategies involving chemical and immunological treatments for the increasingly critical issue of drug‐resistant TB and other forms of TB. Finally, we summarized TB prevention, diagnosis, and treatment challenges and put forward future perspectives. Overall, these findings provide valuable insights into the immunological aspects of TB and offer new directions toward achieving the WHO's goal of eradicating TB by 2035.

## Graphical abstract

Currently, controlling the source of infection, interrupting transmission pathways, and protecting susceptible populations are key measures in tackling MTB infection. However, preventative, diagnostic, and therapeutic interventions for TB are also important in reducing the burden of the disease. First, in terms of preventing MTB infection, the Bacillus Calmette‐Guérin vaccine, the only known vaccine with some protective effects, has limited efficacy and duration of protection in adults. Therefore, the development of multiple vaccines with preventative efficacy may offer new strategies for susceptible populations. Second, in the diagnosis of MTB infection, current diagnostic technologies lack high specificity and sensitivity, and cannot differentiate LTBI from ATB. Therefore, the identification of biomarkers related to immune mechanisms for diagnostic purposes may be an important goal in improving tuberculosis diagnosis efficiency. Last, in the treatment of MTB infection, challenges arise from the effectiveness of conventional chemotherapy drugs and the emergence of MDR‐TB. Thus, the use of immunomodulatory cytokines, immune checkpoint inhibitors, and therapeutic vaccines in combination with anti‐TB chemotherapy drugs may represent effective approaches for TB treatment.

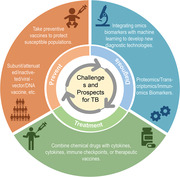

## 1 INTRODUCTION

*Mycobacterium Tuberculosis* (MTB) is a rod‐shaped obligate aerobic bacterium.^1^ MTB cells are flagellum free, but have pili and a thin capsule. The bacterial cell wall lacks phosphoric acid from Gram‐positive bacteria and lipopolysaccharide (LPS) from Gram‐negative bacteria, but it stains positively with acid‐fast staining.^2^ MTB primarily infects humans through airborne transmission, leading to tuberculosis (TB). TB is an infectious disease that seriously threatens human life and health.

The history of human MTB infection can be traced back to the Stone Age, around 7000 years ago. However, it was not until 1882 that German bacteriologist Robert Koch (1843–1910) discovered and proved that MTB is the pathogen responsible for human TB.^2^, ^3^ Subsequently, vaccines and antibiotics were invented, making significant progress in preventing and treating TB. However, TB remains a major infectious disease leading to human deaths.^4^

According to the estimation of the World Health Organization (WHO), the number of newly diagnosed TB cases worldwide in 2021 was estimated to be 10.6 million (95% CI: 9.9–11.0 million), including 6 million adult males, 3.4 million adult females, and 1.2 million children.^3^ It is concerning that the TB incidence rate (number of new cases per 100,000 population per year) increased by 3.6% between 2020 and 2021. At the same time, there has been an increase in the number of TB deaths globally from 2019 to 2021, breaking the previous trend of declining TB incidence and mortality rates.^5^ Unfortunately, the incidence of drug‐resistant tuberculosis (DR‐TB) also increased during the same period. In 2021, there were 450,000 cases of rifampicin‐resistant tuberculosis (RR‐TB, 95% CI: 399,000–501,000).^5^, ^6^ Furthermore, the incidence of TB varies across different regions, with the majority of cases concentrated in the Southeast Asia region, including India, Indonesia, China, the Philippines, Pakistan, Nigeria, Bangladesh, and the Democratic Republic of the Congo, accounting for more than two‐thirds of the global TB burden.^5^ Despite the severity of the TB burden, the global treatment success rate for newly diagnosed TB patients is only 86%, and the treatment success rate for DR‐TB is as low as 60%.^5^

The outcome of MTB infection is closely related to the strength of the host's immune system. The human immune system plays a crucial role in immune surveillance, defense, and regulation, recognizing and eliminating invading pathogens and viruses to maintain homeostasis.^7^, ^8^, ^9^ The immune response to MTB antigens can be divided into two categories: innate immunity (also known as a nonspecific or natural immune response) and adaptive immunity (also known as an acquired or specific immune response). When the host is infected with MTB, the innate immune response and adaptive immune response collaborate to counteract the virulence of MTB. This immune response and interaction with MTB lead to different outcomes of MTB infection in the host.^10^, ^11^ This immunological mechanism provides a solid foundation for the study of TB.

This review aims to analyze the interaction and mechanisms between MTB and the host from an immunological perspective, including the host's immune response to MTB infection and MTB's immune evasion mechanisms. Given the limitations of current diagnostic methods (including sputum examination, sputum culture, tuberculin skin test [TST], and interferon‐gamma release assays [IGRAs], etc.) and treatment regimens (combination chemotherapy with various chemical drugs) for TB, this review also focuses on exploring the application value of biomarkers in the diagnosis and treatment of TB. Based on this, this review will discuss the strategies for chemical treatment and immunotherapy of TB. Finally, this review also addresses the challenges and future directions in preventing, diagnosing, and treating TB from three dimensions. This review aims to support the realization of the WHO's 2035 End TB Strategy, focusing on the prevention, diagnosis, and treatment of TB from an immunological perspective.

## 2 IMMUNOLOGIC MECHANISMS OF MTB–HOST INTERACTIONS

Since the discovery of MTB by Robert Koch under the microscope, our understanding of TB has evolved through three stages: the anatomical stage, the pathological stage, and the immunological stage.^8^, ^9^ Increasing evidence suggests that TB is not only an infectious disease but also an immune‐mediated disease. The most substantial evidence is that over 90% of individuals infected with MTB enter a latent infection state, with only around 10% progressing to active tuberculosis (ATB). Moreover, 85−95% of newly diagnosed cases of ATB originate from immunocompromised individuals who were previously latently infected.^12^ This highlights the crucial role of MTB and host interaction in determining disease outcomes. In the following sections, we will explore the immunological mechanisms of the host's immune response to MTB infection and the immune evasion strategies employed by MTB, considering both perspectives of the host–pathogen interaction.

### 2.1 Host immune response to MTB infection

**FIGURE 1:**
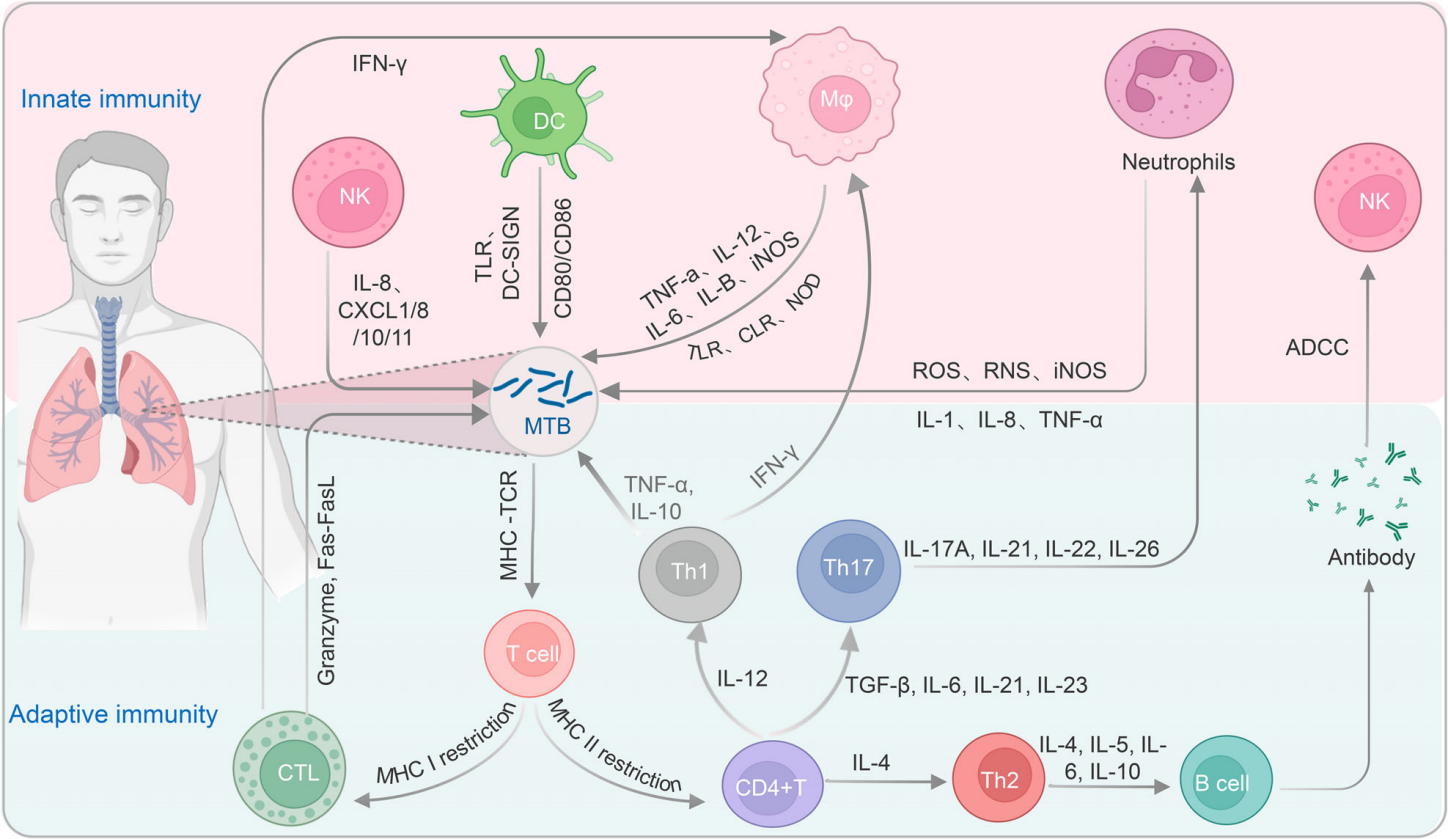
Immune response against MTB infection. Upon entry of MTB through the respiratory tract into the lung tissue, neutrophils, macrophages, NK cells, and DCs within the host are rapidly activated and recruited to the site of infection. While MTB is cleared by these phagocytic cells through ingestion and the release of antimicrobial substances (such as TNF‐α, IL‐12, IL‐10, IL‐6, and iNOS), it is also captured by antigen‐presenting cells (APCs) such as DCs and B cells, which process and present the antigens to T cells, resulting in T cell activation. Activated CD4^+^ T cells can differentiate into different subsets of helper (Th) cells to mediate apoptosis of MTB‐infected cells, while activated CD8^+^ T cells (also known as cytotoxic T lymphocytes, CTLs) can induce the clearance of MTB through pathways involving granule enzymes or the Fas/FasL pathway. MTB, *Mycobacterium tuberculosis*; NK cells, natural killer cells; DCs, dendritic cells; TNF‐α, tumor necrosis factor alpha; IL, interleukin; iNOS, inducible nitric oxide synthase; APCs, antigen‐presenting cells; CTL, cytotoxic T lymphocytes; IFN‐γ, interferon‐gamma; MHC, major histocompatibility complex; ROS, reactive oxygen species; RNS, reactive nitrogen species; TGF‐β, transforming growth factor beta; TLR, Toll‐like receptors.

Following MTB infection, the host typically exhibits one of the following three outcomes: approximately 5% of infected individuals can eradicate MTB from their bodies ultimately, another 5–10% may progress to ATB, while approximately 90% develop latent tuberculosis infection (LTBI), harboring the pathogen long‐term without complete clearance.^13^, ^14^ The factors contributing to these outcomes involve both host and bacterial aspects.^11^ Among them, changes in the host's immune status are the most direct cause of MTB infection progression, while the virulence and invasiveness of MTB are among the major factors influencing the course of infection.^10^, ^11^ Upon entry into the body, innate immune cells such as neutrophils, macrophages, natural killer (NK) cells, and dendritic cells (DCs) rapidly initiate immune response mechanisms, working in coordination with adaptive immune cells including CD4^+^ T cells, CD8^+^ T cells, and B cells, to mount an immune defense (Figure 1).

#### 2.1.1 Nonspecific immune response

The innate immune response is the first line of defense against invading pathogens in a nonspecific manner. It initiates and participates in the adaptive immune response, which enables the body to mount a specific immune response against particular pathogens. The protective function of innate immunity is mediated by the innate immune system, which includes innate immune barriers, innate immune cells, and innate immune molecules. Among them, innate immune cells consist of phagocytes, DCs, NK cells, NK T cells, γδ T cells, and B1 cells, and they act as the primary effectors of innate immune responses.^15^, ^16^

##### 2.1.1.1 The immunologic role of neutrophils and their mechanism of killing MTB

Neutrophils, also known as polymorphonuclear leukocytes, are one of the most abundant types of granulocytes. They are often referred to as professional phagocytes and monocyte–macrophages and play a role in chemotaxis and phagocytosis.^17^ When MTB enters the alveoli through the respiratory tract, neutrophils are among the earliest cells recruited to the site of infection^18^ and form the first line of defense against MTB infection.^19^ Specifically, neutrophils are first released from the bone marrow into the bloodstream and then enter peripheral tissues under the influence of chemotactic stimuli produced by the host or the pathogen through interactions involving the selectin family of C‐type lectin glycoproteins.^20^ Once at the site of infection, neutrophils directly recognize and engulf MTB by interacting with the pathogen's innate components, such as LPS, lipoproteins, lipoteichoic acid, and flagella, through surface receptors (such as TLR1, TLR2, TLR4‐10, and others).^21^ Neutrophils employ both oxygen‐dependent and oxygen‐independent killing pathways to eliminate engulfed MTB. The oxygen‐dependent killing pathway primarily involves reactive oxygen species (ROS) and reactive nitrogen species (such as nitric oxide and peroxynitrite).^22^, ^23^ Upon engulfment of MTB by neutrophils, released ROS and induced nitric oxide synthase can kill the bacteria.^24^, ^25^, ^26^ In the oxygen‐independent killing pathway, neutrophil granules also play a crucial role in inhibiting or killing MTB.^27^ Cationic proteins, defensins, and permeability proteins are the major bactericidal substances, working by direct contact with MTB to eliminate it.^28^ Furthermore, activated neutrophils secrete various chemotactic factors, such as IL‐8, CXCL8, CXCL1, CXCL10, and CXCL11, which amplify their own recruitment and directed chemotaxis, synergistically working with other immune cells to enhance phagocytosis and bactericidal functions, thus enhancing the host's killing effect on MTB.^29^, ^30^

##### 2.1.1.2 The immunologic role of macrophages and their mechanism of killing MTB

Macrophages are a type of white blood cell derived from monocytes and widely distributed throughout the body in various tissues. They function to phagocytose cellular debris and pathogens in both fixed and free forms.^31^, ^32^, ^33^ During MTB infection, resident macrophages in the lungs, also known as lung macrophages, are the primary cell type that initially takes up MTB. MTB activates macrophages by interacting with various pattern recognition receptors (PRRs) on their surface, thereby exerting an anti‐TB effect.^34^ First, surface receptors on macrophages, such as toll‐like receptor 2 (TLR2), TLR4, NOD2, and Dectin‐1, recognize the innate components of MTB, such as glycolipids, peptidoglycan, and other components, leading to the release of anti‐MTB cytokines (such as TNF‐α, IL‐12, IL‐1β, etc.) by macrophages.^35^, ^36^, ^37^, ^38^, ^39^ The actions of these receptors facilitate the clearance of MTB by macrophages. Second, once macrophages engulf MTB, it is degraded by intracellular acid hydrolases.^40^ The phagocytosis of MTB by macrophages also triggers a respiratory burst, producing a series of anti‐MTB effector molecules, such as reactive oxygen intermediates and reactive nitrogen intermediates (RNIs).^41^ H_2_O_2_, an intermediate of reactive oxygen intermediates, is one of the earliest molecules recognized to mediate MTB clearance.^42^, ^43^ Last, different subtypes of macrophages play important roles in the clearance of MTB.^44^, ^45^ For example, M1 macrophages, primarily induced by bacterial LPS and interferon‐gamma (IFN‐γ), release pro‐inflammatory cytokines (such as TNF‐α, IL‐1, and IL‐6) and induce iNOS to kill MTB.^46^ These macrophage subsets' functions are associated with cytokine release and play a crucial role in immune balance. In summary, macrophages play a critical immunological role in MTB infection by recognizing MTB, releasing cytokines, phagocytosing and killing bacteria, as well as interacting with other immune cells to promote the immune response against MTB. In addition, NK cells can also rapidly arrive at the site of MTB infection and participate in the immune response together with macrophages.

##### 2.1.1.3 The immunologic role of NK cells and their mechanism of killing MTB

NK cells are considered a unique type of lymphocyte derived from bone marrow lymphoid progenitor cells and serve as the first line of defense in clearing infected and tumor cells. As a key effector cell of innate immunity, NK cells possess distinctive characteristics such as major histocompatibility complex (MHC)‐unrestricted recognition, antibody independence, and rapid recruitment to infection sites. In humans, mature NK cells can be classified into different subsets based on surface antigens.^47^ Peripheral blood NK cells, for example, express low levels of CD56 and the IgG Fc receptor FcγRIII (CD16), while CD56 high NK cells are predominantly found in lymph nodes.^48^ CD56^high^ NK cells display constitutive high and intermediate affinity IL‐2 receptors and demonstrate the ability to produce cytokines, whereas CD56^low^ NK cells express high levels of CD16 and exhibit potent cytotoxicity.^49^ The specific mechanisms underlying NK cell activities are as follows: First, NK cell function is regulated by a balance of inhibitory signals transmitted by surface inhibitory receptors and activating signals transmitted by activating receptors, as well as the coordination of cytokine signaling. Upon infection, a decrease in MHC class I molecules, which bind to the inhibitory receptors on NK cells, allows recognition and subsequent attack of the MTB by NK cells.^50^ Second, activating receptors on the surface of NK cells, such as NKp44, NKp46, and NKp30, can directly bind to various cell wall components of MTB, such as arabinogalactan–peptidoglycan, mycolic acids, and arabinogalactan derivatives. This leads to the release of IFN‐γ and IL‐22 by NK cells, which serve to inhibit or eliminate MTB.^51^, ^52^ Finally, activated NK cells can nonspecifically recognize infected target cells and induce target cell apoptosis through granule exocytosis pathways (such as perforin and granzymes) and death receptor pathways (primarily the Fas/FasL pathway).^53^ Additionally, NK cells can also be directly activated through antibody‐dependent cell‐mediated cytotoxicity, leading to the secretion of various cytokines and chemokines, as well as the modulation of various effector cells, thereby influencing the adaptive immune response. In summary, NK cells' mechanisms of action impact MTB's survival within granulomas and regulate multiple immune effector cells, thereby affecting the host's adaptive immune response. This parallel with DCs demonstrates their ability to shape the immunological microenvironment and modulate the functions of different effector cells, ultimately aiding in the host defense against MTB.

##### 2.1.1.4 The immunologic role of DCs and their mechanism of killing MTB

DCs are a subset of highly efficient antigen‐presenting cells (APCs) originating from myeloid progenitors. They play a major role in antigen uptake, processing, and presentation. DCs are currently the only APCs capable of activating naïve T cells, thereby initiating adaptive immune responses. They serve as a crucial link between innate and adaptive immunity.^54^ DCs are widely distributed in human tissues, except for the brain, and can be classified into immature DCs (iDCs) and mature DCs (mDCs) based on their stage of development.^55^ On one hand, during MTB infection, iDCs utilize PRRs (such as TLR2 and TLR4, and DC‐SIGN) on their surface to mediate the internalization of MTB antigens and subsequent phagocytosis of MTB.^56^ After phagocytosing MTB, iDCs upregulate the chemokine receptor CCR7, which enables them to migrate from peripheral inflammatory tissues to secondary lymphoid tissues via the bloodstream or lymphatic circulation under the guidance of chemokine receptor CCR7.^57^ It is during this process of migration that iDCs mature. mDCs can then present processed MTB antigens to T cells, thereby initiating a specific immune response. On the other hand, mDCs upregulate the costimulatory molecules CD80/CD86, which activate T cells and promote their differentiation toward CD4+ T cells.^58^ Furthermore, CD4+ T cells, under the influence of cytokines secreted by mDCs (such as IL‐12, TNF‐α, and IFN‐α), differentiate into Th1 cells.^59^ The production of IFN‐γ by Th1 cells can further activate macrophages, enhancing their ability to eliminate MTB. Thus, DCs play a critical role in the collective defense against MTB infection by both innate and adaptive immunity.

#### 2.1.2 Adaptive immune responses

MTB is an intracellular pathogen, and the immune response against this intracellular pathogen mainly relies on specific immunity. It is well known that specific immunity in the body is generally divided into cellular immunity and humoral immunity. Cellular immunity refers to the immune responses mediated by T cells, while humoral immunity is usually mediated by B cells. Among them, mature T cells can be further differentiated into two subtypes, CD4^+^ T cells and CD8^+^ T cells, based on the different surface markers they express.^60^

##### 2.1.2.1 The immunologic role of T lymphocytes and their mechanism of killing MTB

T‐cell immunity refers to the immune response mounted by the body following stimulation by MTB. This process involves the initial processing, presentation, and recognition of MTB antigens by APCs, which in turn triggers a series of immune reactions, including T‐cell activation, proliferation, and differentiation, leading to the elimination of the invading foreign antigens. The specific mechanisms of this process are as follows:

First, mDCs present antigen peptides bound to MHC molecules on their surface. This MHC–antigen peptide complex can interact with the T cell receptor (TCR) on the surface of T cells, thereby inducing T‐cell activation.^61^ Interestingly, the TCR selectively recognizes and accepts different MHC–antigen peptide complexes presented by APCs, thereby mediating different immune responses.^62^ For example, T cells expressing CD4 primarily interact with MHC class II molecules, while T cells expressing CD8 primarily interact with MHC class I molecules.

Second, after the binding of the TCR to the MHC–antigen peptide complex, signals for antigen recognition are transmitted into the T cell, initiating T‐cell activation. In parallel, the interaction between costimulatory molecules on the surface of APCs and costimulatory receptors on the surface of T cells generates costimulatory signals that synergize with the primary activation signal to activate T cells.^63^

Last, activated T cells can differentiate into effector cells with different functions under the influence of various cytokines. On one hand, the expression of MHC class II molecules on APCs can induce initial CD4^+^ T cells (Th0) to differentiate into different subsets of helper T cells, such as Th1, Th2, Th17, and Treg cells.^64^ Among them, Th1 cells are the main immune cells against MTB. They primarily secrete cytokines such as IFN‐γ, IL‐2, and TNF‐α.^65^ These cytokines recruit monocytes and neutrophils, positively regulate the direct cytotoxicity of macrophages, and induce the synthesis of inflammatory mediators and reactive oxygen and nitrogen species,^66^, ^67^ thereby facilitating the elimination of MTB. Th17 cells, on the other hand, mainly combat MTB by enhancing host defense. They secrete cytokines such as IL‐17A, IL‐21, IL‐22, and IL‐26 to recruit neutrophils to the site of infection, thereby enhancing the inflammatory response.^68^, ^69^

On the other hand, CD8^+^ T cells recognize antigen peptides presented by MHC class I molecules, which leads to their own proliferation and differentiation into cytotoxic T lymphocytes (CTLs). CTLs can then lyse intracellularly infected MTB cells and secrete cytokines.^70^ The pathways by which CTLs lyse infected MTB cells include: (1) CTLs bind to antigen complexes on the surface of infected MTB cells, allowing perforin and granulysin from the CTLs to enter the immunological synapse through exocytosis, disrupting the normal osmotic gradient of the infected cells and causing them to lyse, after which MTB can be phagocytosed and eliminated by surrounding macrophages.^70^, ^71^ (2) Activated CTLs express Fas ligand (FasL), which can bind to Fas receptors on infected MTB cells, initiating apoptotic signals and activating the caspase‐8‐mediated apoptotic signaling pathway, leading to the apoptosis of infected MTB cells.^72^ (3) Activated CTLs can secrete the cytokine IFN‐γ, which positively regulates the expression of ROS and RNIs in monocytes and macrophages, thereby enhancing their cytotoxicity.^73^, ^74^

Therefore, T‐cell immune responses play a crucial role in the body's defense against MTB infection and are also the primary target for strategies in TB vaccine development.

##### 2.1.2.2 The immunologic role of B lymphocytes and their mechanism of killing MTB

In the defense and immune response against MTB infection, it is not only specific cellular immunity or humoral immunity that plays a role, but rather the interaction of all immune cells in the immune system. This challenges the earlier notion that B cells do not significantly combat MTB infection.^75^, ^76^

In the immune response against MTB, B cells primarily produce antibodies and release toxins to destroy infected cells.^77^ Specifically, B cells, as specialized APCs, are activated after the uptake of antigens through surface receptors. They present the antigens and stimulate the activation of CD4^+^ T cells. Activated CD4^+^ T cells can produce different cytokines and differentiate into Th1 and Th2 helper T cells under their regulation, modulating B lymphocyte antibody response.^78^, ^79^ This process helps mediate the apoptosis of MTB‐infected cells.

Furthermore, B cells can differentiate into different subsets, including B effector 1 (Be1), B effector 2 (Be2), and B regulatory cells (Breg).^80^ Be1 and Be2 cells can produce different cytokines to induce the development of initial CD4^+^ T cells into effector Th1 and Th2 T cells, which are involved in the clearance of MTB from the body.^81^

Last, antibodies are crucial in defending against pathogen invasion and neutralizing microbial toxins. The mechanism by which antibodies regulate antigen presentation through Fcγ receptors (FcR) has been considered a potential approach for vaccination.^82^ Although the exact mechanism of antigen presentation regulated by FcR is not fully understood, FcR is an essential immunoregulatory molecule.^83^ FcR includes FcRI (CD64), FcRII (CD32), and FcRIII (CD16), each with different intracellular motifs (ITAM or ITIM) that divide the receptors into inhibitory and activating types.^84^ These receptors participate in the complex activation of T cells by inhibiting or promoting the maturation and antigen presentation processes of DCs.^85^ Studies, such as those conducted by Typiak et al.^82^ in patients with pulmonary granulomas and TB, have demonstrated the role of Fc receptors in the context of MTB infection. Therefore, B cells' contribution to host defense against MTB infection should not be overlooked.

### 2.2 Immune escape mechanism of MTB

**FIGURE 2:**
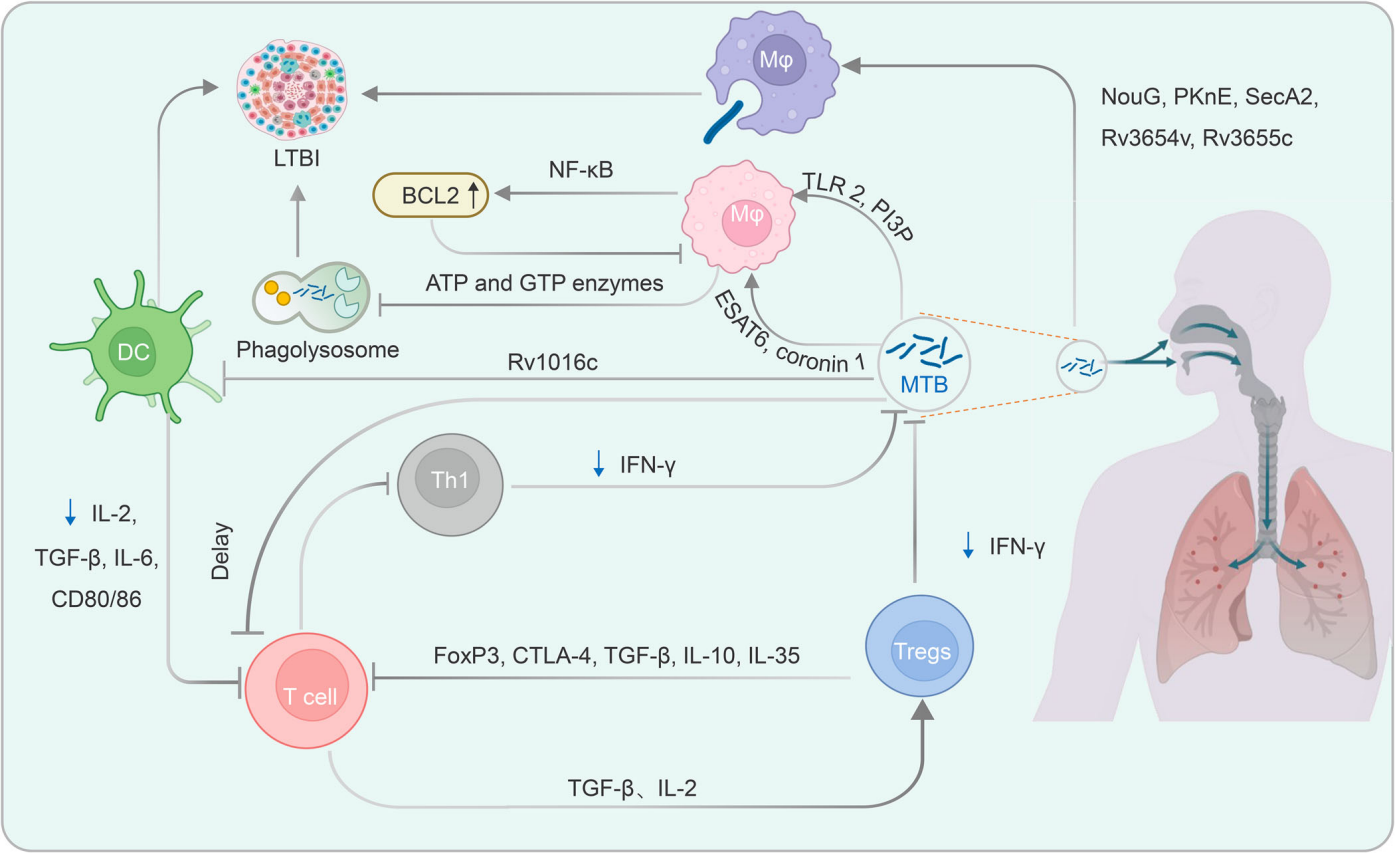
Immune evasion mechanisms of MTB. During the host's defense against MTB infection, MTB also exerts counteraction against the host's immune system, leading to the development of LTBI or ATB. First, MTB can inhibit apoptosis of macrophages by secreting early antiapoptotic proteins such as NouG, PKnE, and SecA2, thereby maintaining its survival within macrophages. Additionally, MTB activates the NF‐κB pathway by binding to TLR2 on the surface of macrophages, upregulating the expression of antiapoptotic protein BCL2 and inhibiting macrophage apoptosis. Second, infected cells manipulate PI3P and LprE to suppress macrophage autophagy, enabling immune evasion of MTB. Last, MTB reduces the antigen presentation capacity of APCs through its component Rv1016c, thereby inhibiting T cell activation. Additionally, prolonged stimulation by MTB increases the proportion of CD4^+^ T cells differentiating into regulatory T cells (Tregs), which express inhibitory proteins on their cell surface that bind to TCR, ultimately leading to functional exhaustion of T cells. MTB, *Mycobacterium tuberculosis*; LTBI, latent tuberculosis infection; ATB, active tuberculosis; TLR, Toll‐like receptors; PI3P, phosphatidylinositol 3‐phosphate; APCs, antigen‐presenting cells; Treg, regulatory T lymphocytes; TCR, T cell receptor; TGF‐β, transforming growth factor beta; IL, interleukin; IFN‐γ, interferon‐gamma.

The war between MTB and humans has been ongoing for thousands of years. Throughout the lengthy process of evolution, MTB has developed various mechanisms to evade the immune cells of its host. These evasion strategies prevent the host's immune system from effectively monitoring and combating MTB, leading to the development of LTBI or ATB. The strategies employed by MTB to evade immune surveillance, recognition, and clearance can generally be categorized into three types: intrinsic virulence factors of MTB, evasion of innate immunity, and evasion of adaptive immunity^86^, ^87^ (Figure 2).

#### 2.2.1 Virulence factors of MTB

Virulent strains of MTB can secrete specific antiapoptotic genes or components (such as the type I NADH dehydrogenase NuoG, serine‐threonine kinase PknE, secA2, Rv3654c, and Rv3655c) to inhibit apoptosis of macrophages, thereby reducing the bactericidal effect of MTB and promoting its coexistence with the host.^88^, ^89^ These secreted proteins from MTB can transmit external signals of the bacteria to the cytoplasm and subsequently inhibit apoptosis of immune cells by blocking signaling pathways. For example, Riendeau et al.^90^ found that the weakly virulent strain MTB H37Rv and *Mycobacterium bovis* Bacillus Calmette‐Guérin (BCG) could induce strong apoptosis in THP‐1 cells differentiated by phorbol‐12‐myristate‐13‐acetate. This further demonstrates that MTB's intrinsic virulence can regulate host macrophages' apoptosis response.

#### 2.2.2 Evasion of nonspecific immune response

In the innate immune evasion mechanisms of MTB, inhibition of macrophage apoptosis and autophagy is crucial. First, the structural characteristics of MTB and its secretion of antiapoptotic protein genes can suppress the maturation and acidification of phagosomes and lysosomes in macrophages. For example, early secreted antigen target 6 (ESAT6), culture filtrate proteins (secreted ATPase1/2 and secA1/2), and tryptophan aspartate rich coat protein (coronin 1) in phagosomes containing live MTB can lower the cell pH by inhibiting intracellular ATP and GTPases, thus inhibiting the maturation of phagolysosomes within macrophages.^91^, ^92^ The expression level of coronin 1 is positively correlated with the amount and activity of intracellular MTB, and MTB can upregulate the expression of coronin 1 on macrophage membranes to suppress phagolysosome formation, promoting coexistence between MTB and host macrophages.^93^

Second, preventing the fusion of phagosomes with lysosomes is an important mechanism for inhibiting macrophage apoptosis and autophagy. Studies have reported that the LPSs of MTB can induce activation of NF‐κB by binding to TLR2 on the surface of macrophages.^94^ The activated NF‐κB signaling pathway upregulates the expression of the antiapoptotic factor Bcl2, thereby preventing the fusion of phagosomes with lysosomes within macrophages and resulting in immune evasion by MTB.^95^ Furthermore, the regulation of conversion between host phosphatidylinositol components and the replacement of different Rab family members on MTB phagosomes are mechanisms for MTB survival and growth within cells. Phosphatidylinositol 3‐phosphate (PI3P) is a critical factor for forming phagolysosomes.^96^ After infection with MTB, PI3P reduces the recruitment and association with endosomal membrane effectors (e.g., EEA1) necessary for binding early phagosomes within macrophages.^97^ This inhibits the fusion process between phagosomes and lysosomes within macrophages, thereby preventing autophagy.

Last, MTB can also suppress macrophage autophagy and diminish the host's ability to clear MTB by regulating IL‐6 and LprE lipid protein.^98^, ^99^ LprE protein primarily inhibits the expression of vitamin D3 (cathelicidin inducer) through the TLR2 signaling pathway, thereby interfering with vitamin D3‐mediated immune responses.^100^ Studies have shown that the deletion of LprE in MTB significantly affects the survival rate of MTB within macrophages.^99^ These findings highlight the close relationship between MTB's evasion of innate immunity and the inhibition of macrophage apoptosis and autophagy. Unfortunately, MTB cannot only evade innate immune responses but also adaptive immune responses.

#### 2.2.3 Evasion of adaptive immune responses

During the host defense against MTB infection, CD4^+^ T cells and CD8^+^ T cells are the main effectors of specific immunity (as mentioned earlier). However, T cells also play important roles in the process of MTB immune evasion. First, certain proteins secreted by MTB can inhibit the function of DCs by inducing anti‐inflammatory cytokines or interfering with the presentation of MTB antigens to T cells,^101^, ^102^ thereby suppressing T cell activation and facilitating immune evasion by MTB. For example, Su et al.^103^ found that high expression of the mannose‐capped protein Rv1016c in MTB‐BCG vaccine (rBCG‐Rv1016c) reduced the production of cytokines IL‐2, TGF‐β, IL‐6, and costimulatory molecules CD80 and CD86 by DCs in a mouse model, thereby affecting T cell activation.

Second, CD4^+^CD25^high^ regulatory T cells (Tregs) can be activated and expanded during chronic and long‐term stimulation by MTB. The highly expressed immunosuppressive proteins on the surface of Tregs, such as Foxp3, cytotoxic T‐lymphocyte antigen 4 (CTLA‐4), and CCR4, can interact with TCR, leading to immune suppression and prolonged persistence of MTB within host cells.^104^, ^105^ Additionally, studies have found that the removal of Treg cells can increase the secretion of IFN‐γ,^106^ suggesting that the expansion of Treg cells can affect the anti‐TB function of IFN‐γ.

Finally, it has been observed that the response of CD4^+^ T cells and CD8^+^ T cells to MTB infection exhibits relatively delayed kinetics compared with acute viral infections or other intracellular bacterial infections.^107^ For instance, Urdahl et al.^108^ proposed that this delay may be attributed to the transport of MTB from the lungs to the lymph nodes and the suppression of T cell priming by Treg cells. In conclusion, the mechanisms employed by MTB to evade adaptive immune responses are complex and diverse.

## 3 BIOMARKERS FOR TB DIAGNOSIS AND TREATMENT

TB remains a significant burden of infectious disease globally, especially in developing countries and extremely underdeveloped regions. Therefore, early diagnosis and treatment of TB are crucial in reducing the incidence and mortality rates of TB.^109^ However, the current available diagnostic technologies are insufficient to achieve effective, rapid, and accurate diagnosis of TB. As a result, there is an urgent need for a simple, highly sensitive, and highly specific diagnostic method. Current clinical tests face certain issues, such as long testing cycles and inadequate sensitivity in sputum examination, lower specificity of GeneXpert MTB/RIF (gene amplification technology), inability to differentiate TB from other infections through imaging alone due to similar manifestations, and poor specificity of the TST and IGRAs in immunocompromised individuals.^110^

Fortunately, in early disease research, multiomics technologies have been widely applied to identify novel biomarkers and anti‐TB drugs. Multiomics primarily utilizes high‐throughput methods to rapidly obtain hundreds or thousands of biomarkers, including DNA, RNA, proteins, and metabolites, namely genomics, transcriptomics, proteomics, and metabolomics.^111^, ^112^ These biomarkers play a significant role in combating TB, and the use of reliable biomarkers helps clinicians make prompt decisions and optimize treatment timing and opportunities. This chapter focuses on the application of current multiomics biomarkers in the early diagnosis and treatment of TB, offering new directions and approaches in the search for novel biomarkers and anti‐TB drugs. However, the application and research for each specific biomarker are still in continuous development. Therefore, there is currently no universally applied biomarker for diagnosing and treating TB. Further research and clinical validation are required to identify reliable biomarkers and implement them in real clinical practice. These efforts improve TB's diagnostic accuracy and treatment effectiveness while reducing its transmission and incidence rates.

Table 1 outlines some potential multiomics biomarkers currently under investigation, although it does not imply their widespread application in clinical practice. The diagnosis and treatment of TB involve a complex process that requires the comprehensive consideration of multiple factors and the integration of various diagnostic techniques and methods. Using multiomics technologies provides new directions and possibilities for TB diagnosis research. However, further efforts and research are needed to make it an effective tool in clinical practice.

**TABLE 1:** Biomarkers for TB diagnosis and treatment that are currently in the research phase.

Types of research	Sample	Biomarker	Sensitivity	Specificity	Other performance	Monitoring periods of infection	References
Protein markers	94 HC, 93LTBI, 92 ATB	Rv1408+R0248+Rv2026c+Rv2716+Rv2031c+Rv2928+Rv2121c	93.3%	93.1%	NA	NA	^113^
	100ATB, 100LTBI, 100HC	Rv1860, RV3881c, Rv2031c, and Rv3803c	93.3%	97.7%	NA	NA	^114^
	Recent LTBI (*n* = 13) or distant LTBI (*n* = 12) LTBI, dormant (*n* = 19), or active TB (*n* = 15)	MDP‐1, ESAT6, antigen 85A, Acr	NA	NA	MDP‐1, ESAT6 antibody titre positive 80% at ATB, 77% in LTBI group	Recent LTBI individuals had significantly higher antibody titres to ESAT6, Ag85A, Acr and MDP1 than distant LTBI	^115^
Genetic markers (transcriptomics)	301 ATB, 68 LTBI, and 278 HC	rs9061 in the SP110 gene	NA	NA	NA	Significantly associated with increased LTBI susceptibility	^116^
	51 ATB, 44 LTBI, and 35 HC	TNFRSF3C, EBF10, and A3ML2	86.2%	94.9%	NA	NA	^117^
	97 PTB cases, 140 NTB DC, and 245 HC	Three lncRNAs, ENST00000497872, n333737, and n335265	0.86 at ATB	0.82 at ATB	NA	NA	^118^
	50 ATB, 33 LTBI, and 30 HC	miRNA‐29a‐3p	86.0% at ATB, 84.8% at LTBI	73.0% at ATB, 70.0% at LTBI	NA	NA	^119^
	Discovery: 3 ATB and 3 HC; validation: 40 ATB and 40 HC	Circular RNA hsa_circRNA_001937	85%	77.5%	AUC = 0.873	NA	^120^
Immune markers (cytokines)	ATB 20, untreated LTBI 20, treated LTBI 20, HC 20	CCL1, CXCL10, VEGF, and ADA2	95%	90%	NA	NA	^121^
	28atb, 24ltbi, 26hc	Eotaxin, MDC, MCP‐1	87.76%	91.84%	NA	NA	^122^
	2836 subjects, 3219 blood samples	IP‐10	0.86	0.88	NA	NA	^123^

### 3.1 Protein biomarkers

The expression profile of proteins in MTB differs during the latent, replicative, and active phases, leading to the increasing application of proteomics in diagnosing and monitoring TB. MTB proteins are typically differential antigens, while host proteins are mainly cytokines. Therefore, the host's immune response to antigens varies at different stages of infection. For example, MTB‐specific antigens such as ESAT‐6 and CFP‐10 are absent in BCG strains, and detecting specific immune responses to these antigens can differentiate MTB infection from vaccine reactions.^113^ Despite the favorable sensitivity and specificity demonstrated by newly developed skin tests, they still cannot distinguish between ATB and LTBI. Therefore, efforts have been focused on developing novel protein biomarkers to improve the diagnosis and monitoring of MTB infection.

A study based on microarray technology used 4262 antigens from MTB to differentiate between ATB and LTBI in serum samples from individuals. Candidate antigens were validated by enzyme‐linked immunosorbent assay (ELISA), and eventually, a combination of antigens Rv1408+R0248+Rv2026c+Rv2716+Rv2031c+Rv2928+Rv2121c was determined, with a sensitivity of 93.3% and specificity of 93.1% in distinguishing ATB from LTBI.^114^ Another study utilizing microarray technology constructed 64 MTB‐related antigens and identified a combination of four proteins (Rv1860, Rv3881c, Rv2031c, and Rv3803c) as the optimal biomarker panel, achieving sensitivities of 93.3 and 97.7% in distinguishing ATB and LTBI, respectively.^115^ Furthermore, a single‐center prospective study demonstrated that the DNA‐binding protein of MTB (MDP‐1) could serve as a potential biomarker for distinguishing LTBI from ATB (80% positive antibody titer in ATB patients vs. 77% in the LTBI group), as well as monitoring the efficacy of anti‐TB treatment.^116^ Protein transcriptomic technology, as an emerging technique integrated with machine learning, has shown promising diagnostic potential and is expected to become a reliable biomarker for the future diagnosis and treatment of MTB infection.

### 3.2 Genetic biomarkers

In the late 20th century, researchers began to understand the genome of MTB comprehensively.^117^ Transcriptomics can reveal the expression profiles of genes, including biomarkers that help understand the interaction between MTB and the host, including noncoding RNAs. For instance, the SP110 gene significantly regulates the innate immune response to MTB infection. A genomic study involving individuals from the Taiwanese population investigated 301 patients with ATB, 68 with LTBI, and 278 healthy controls.^118^ The study found a significant association between the rs9061 variant in the SP110 gene and increased susceptibility to LTBI.^118^ Another transcriptomic study used unsupervised classification to identify differentially expressed genes among individuals with ATB, LTBI, and healthy controls.^119^ The study validated TNFRSF3C, EBF10, and A3ML2 as a biomarker combination that achieved a sensitivity of 86.2% and specificity of 94.9% in classifying these three groups.^119^

MicroRNAs (miRNAs) in transcriptomics are short RNA molecules that regulate the interaction between the host and pathogens, including MTB.^124^ In a recent case‐control study, miRNA‐29a‐3p was identified as a sensitive biomarker for active pulmonary tuberculosis (PTB), with a sensitivity of 86.0% and specificity of 73.0% and showed a sensitivity of 84.8% and specificity of 70.0% in LTBI.^121^ Additionally, long noncoding RNAs (lncRNAs), which are noncoding RNA molecules longer than 200 nucleotides, play a vital role in transcriptional regulation, post‐translational modification, and epigenetic regulation of gene expression.^122^ Researchers have discovered that three lncRNAs (ENST00000497872, n333737, and n335265) showed significant differential expression in patients with ATB compared with healthy individuals.^123^ These lncRNAs hold potential as biomarkers for clinical diagnosis of TB patients, displaying a sensitivity of 0.86 and specificity of 0.82 in distinguishing TB patients from nontuberculous disease (NTB).^123^ Furthermore, circular RNAs (circRNAs), known for their stability, are gene expression regulators involved in the interaction of cytokines and chemokines during MTB infection.^125^ One study found that the expression of hsa_circRNA_001937 was upregulated, while hsa_circRNA_102101 was downregulated during lung infection with MTB.^126^

In summary, omics‐based biomarkers help us understand the gene expression profiles at different infection stages, aiding in the diagnosis and monitoring of MTB infections. In the future, these omics‐based biomarkers hold promise to become valuable tools in the diagnosis and treatment of MTB infections, offering new methods and insights.

### 3.3 Immune‐related biomarkers

After being infected with MTB, the human body secretes different types of cytokines, and these cytokines have varying concentrations at various stages of MTB infection.^127^ The diagnosis and treatment of tuberculosis can be assisted by detecting and monitoring the changes in the concentration of these cytokines. Among them, IFN‐γ is the most commonly used cytokine for detecting MTB infection, and TST has been widely used for diagnosing ATB and LTB. However, due to TST's inability to differentiate between MTB infection and infection with other nontuberculous mycobacteria, as well as cases involving BCG vaccination, in recent years, IGRAs based on T‐cell immune responses have become the most popular methods for TB diagnosis.^128^ IGRA measures IFN‐γ production or the quantity of IFN‐γ‐producing T cells in whole blood by stimulating MTB‐specific antigens (such as ESAT‐6 and CFP‐10).^129^, ^130^ However, both TST and IGRA require clinical correlation for diagnosis. Therefore, in terms of cytokines, further research is needed to identify more effective biomarkers to assist in TB diagnosis.

A cross‐sectional study found that a combination of four cytokines (CCL1, CXCL10, VEGF, and ADA2) achieved a sensitivity of 95% and specificity of 90% in differentiating between active and latent TB in a discovery cohort, but the sensitivity and specificity were relatively lower in validation cohorts from different countries.^131^ Another study used the T.SPOT.TB assay to detect peripheral blood samples and found that a combination of three cytokines (eotaxin, MDC, MCP‐1) had an area under the curve of 0.94 in differentiating between ATB and LTB, with sensitivity and specificity of 87.76 and 91.84%, respectively.^127^ Additionally, a review of studies on IP‐10 showed that in a total of 2836 subjects and 3219 blood samples, IP‐10 had a sensitivity of 0.86 (95% confidence interval: 0.80–0.90) and specificity of 0.88.^132^

In conclusion, the accuracy of the final diagnosis may depend on the proper selection of cytokines or combinations thereof. Therefore, a smaller number of cytokines or their combinations, in conjunction with appropriate diagnostic tools, are expected to provide strong support for diagnosing, treating, and monitoring TB in the future.

### 3.4 Role of biomarkers in disease surveillance and treatment decisions

Biomarkers play a crucial role in TB disease surveillance and treatment decisions by providing valuable information about the infection status, disease severity, treatment response, and prognosis of patients. Here is a detailed discussion on the role of biomarkers in TB disease surveillance and treatment decisions:

1. *Diagnosis*: Biomarkers can aid in the accurate and timely diagnosis of TB. Traditional diagnostic methods like sputum smear microscopy and culture have sensitivity and turnaround time limitations. Biomarkers, such as IGRAs and nucleic acid amplification tests, can detect MTB‐specific components or DNA/RNA, providing more sensitive and rapid diagnostic options.^133^, ^134^, ^135^
2. *Treatment response monitoring*: Biomarkers enable the monitoring of treatment response during anti‐TB therapy. They can assess the effectiveness and predict the outcomes of treatment. For example, over time, a decline in the concentration of specific biomarkers, such as C‐reactive protein (CRP) or procalcitonin, may indicate a positive response to treatment.^136^ Serial measurements of these biomarkers can help determine treatment efficacy and guide adjustment if necessary.
3. *Drug resistance detection*: Biomarkers can identify drug‐resistant strains of MTB, which is crucial for selecting appropriate anti‐TB drugs. Molecular assays, like the GeneXpert MTB/RIF assay and innowaveDX MTB/RIF test, target specific genetic mutations associated with drug resistance, rapidly and accurately identifying drug‐resistant strains.^137^, ^138^
4. *Disease severity assessment*: Biomarkers can help evaluate the severity of TB infection and disease progression. Inflammatory markers, such as cytokines (e.g., IL‐6, TNF‐α) and acute‐phase reactants (e.g., CRP), can reflect the extent of inflammation and tissue damage.^139^ High levels of these biomarkers may indicate advanced disease and the need for more aggressive treatment.
5. *Prognosis and risk stratification*: Biomarkers can assist in determining a patient's prognosis and risk of developing severe disease. Certain biomarkers, such as adenosine deaminase or IP‐10, have been associated with disease progression and can help identify patients who require close monitoring or early intervention.^140^
6. *Treatment duration optimization*: Biomarkers can guide the optimal duration of TB treatment. Some biomarkers, like sputum culture conversion, can indicate when the bacteria are no longer viable.^141^ Monitoring this biomarker can help determine the appropriate length of treatment, potentially reducing the duration and associated risks of therapy.
7. *Adverse drug reaction prediction*: Biomarkers have the potential to predict the occurrence of adverse drug reactions (ADRs) during TB treatment. Genetic markers, such as the NAT2 genotype, can identify individuals at higher risk of ADRs, allowing clinicians to adjust treatment regimens accordingly.^142^

Biomarkers play a multifaceted role in TB disease surveillance and treatment decisions. They contribute to accurate diagnosis, treatment response monitoring, drug resistance detection, severity assessment, prognosis determination, treatment duration optimization, and prediction of ADRs. Incorporating biomarker‐based approaches into clinical practice can enhance TB management and improve patient outcomes.

## 4 THERAPEUTIC INTERVENTION STRATEGIES FOR TB

TB is a deadly bacterial infection, and significant progress has been made in its prevention, diagnosis, and treatment since the identification of MTB as the causative agent in 1882.^2^ In recent years, TB incidence and mortality rates have declined due to the development of anti‐TB drugs and improved hygiene and living conditions. However, factors such as drug‐resistant strains, immunosuppressants, drug addiction, poverty, and population mobility have worsened the TB epidemic.^5^

In terms of TB treatment strategies, the primary measure is effective prevention. Currently, the only available vaccine for TB is BCG, but its effectiveness is limited, particularly in adults. The next step is to achieve effective, rapid, and accurate TB diagnosis. However, there is currently a lack of effective methods for differentiating LTBI from ATB, which leads to delays in TB treatment and potential reactivation. Finally, the use of effective treatment methods is crucial. A combination of multiple anti‐TB drugs is the main approach to treating TB. However, long‐term use of multiple drugs can lead to toxicity, poor patient adherence, and the emergence of drug resistance.^143^, ^144^, ^145^ Additionally, inadequate antibiotic management and patient compliance issues have significantly increased cases of multidrug‐resistant TB (MDR‐TB) and extensively DR‐TB (XDR‐TB).^146^ All these factors make TB prevention and control more challenging. Therefore, there is an urgent need to search for new MTB drugs, improve the diagnostic capabilities of TB, and develop novel TB vaccines.

With the rapid development of bioinformatics and immunology, new TB treatment approaches are garnering attention. Among them, the development of TB‐specific immunotherapy can effectively modulate the immune response against TB, providing new avenues for comprehensive treatment and more effective prevention and intervention in high‐risk populations.^145^

### 4.1 Chemotherapy strategies for TB

TB treatment goals extend beyond disease eradication and include preventing long‐term morbidity and adverse reactions. Once diagnosed with TB, treatment should be a public health priority, with the overall goal of eradicating MTB infection.^147^ It is important to note that the treatment focus for ATB is to improve the patient's clinical condition, prevent the development or worsening of drug resistance, and prevent disease relapse. Treating individuals with LTBI is also aimed at preventing LTBI from progressing to ATB.^148^

For centuries, TB treatment methods have primarily involved drug therapy and supportive care, with drug therapy being the main approach. According to the WHO Global TB Report of 2022, 85% of drug‐susceptible TB patients can be successfully treated.^109^ However, the treatment success rate for XDR‐TB is lower (only 57%), and drug resistance is likely a result of multiple factors.^5^ Therefore, this section summarizes existing TB treatment regimens and introduces three new anti‐TB drugs (Table 2).

**TABLE 2:** Chemotherapeutic strategies for tuberculosis.

Organization or countries	LTBI	Sensitive TB	DR‐TB
WHO		2HRZE/4HR treatment for 6 months is the preferred treatment option. 2HRZ(E)/2HR treatment for 4 months (3 months to 16 years of age Children and adolescents with nonsevere TB between and without suspicion or evidence of multidrug/rifampicin‐resistant TB).	BPaLM program (bedaquiline, propranolol, linezolid, and moxifloxacin for 6 months). Nine months of an all‐oral regimen (bedaquiline (used for 6 months), combined with levofloxacin/moxifloxacin, ethionamide, ethambutol, isoniazid (high dose), pyrazinamide, and clofazimine (for 4 months, with the possibility of an extension to 6 months if the patient's sputum smear is still positive at the end of the 4th month); followed by administration of levofloxacin/moxifloxacin, clofazimine, ethambutol, and pyrazinamide (5 The treatment was followed by levofloxacin/moxifloxacin, clofazimine, ethambutol, and pyrazinamide (5 months). Ethionamide may be substituted for 2 months of linezolid (600 mg/d).
USA	Isoniazid plus rifapentine once weekly for 3 months (adults and children over 2 years of age, including HIV‐positive). Rifampicin daily for 4 months (HIV‐negative adults and children of all ages). Daily isoniazid plus rifampicin for 3 months (adults and children of all ages and HIV‐positive people).	Daily treatment program for 4 months (intensive phase, 8 weeks of daily treatment with RPT, MOX, INH, and PZA, followed by a continuous phase, 9 weeks of daily treatment with RPT, MOX, and INH). The preferred regimen for TB treatment in adults is a 2‐month intensive period of isoniazid (INH), rifampicin (RIF), pyrazinamide (PZA), and ethambutol (EMB), followed by a 4/7‐month period of INH and RIF.	
China		In patients with unknown susceptibility or resistance to rifampicin, first‐line antituberculosis drugs (isoniazid, rifampicin, rifapentine, pyrazinamide, ethambutol, and streptomycin) are recommended for antituberculosis treatment, with preference for fixed‐dose combinations, in all special circumstances.	For rifampicin‐resistant patients, the regimen is divided into a long‐course regimen (an 18–20‐month regimen consisting of at least four effective anti‐TB drugs, with standardized regimens recommended for fluoroquinolone susceptibility and resistance, respectively) and a short‐course regimen (a standardized combination regimen consisting of seven anti‐TB drugs over a period of 9–11 months).

Abbreviations: DR‐TB, drug‐resistant tuberculosis; E(EMB), ethambutol; H(INH), isoniazid; LTBI, latent tuberculosis infection; MOX, moxifloxacin.; PZA, pyrazinamide; R, rifampicin; RPT(RIF), rifapentin; USA, United States of America; WHO, World Health Organization; Z, pyrazinamidef.

#### 4.1.1 Chemotherapeutic strategies for LTBI

Approximately one‐fourth of the global population is estimated to be infected with MTB.^149^ The majority of infected individuals exhibit no symptoms and are classified as having LTBI. Without treatment, around 5−10% of LTBI patients will develop TB over their lifetime, emphasizing the importance of treating LTBI to prevent disease progression.^9^, ^150^, ^151^ The Guidelines for the Treatment of Latent Tuberculosis Infection: Recommendations from the National Tuberculosis Controllers Association and CDC guide LTBI treatment.

For LTBI treatment, a preferred regimen involves once‐weekly administration of isoniazid and rifapentine for 3 months, with strong recommendations for adults and children aged 2 years and above, including HIV‐positive individuals (as long as there are no drug interactions). For HIV‐negative adults and children of all ages, the recommended first‐line treatment option is daily administration of rifampicin for a period of 4 months. The daily regimen of isoniazid and rifapentine for 3 months is conditionally recommended as the preferred treatment option for adults and children of all ages, including HIV‐positive individuals. Furthermore, an alternative regimen involving daily administration of isoniazid for 6 or 9 months is strongly recommended for HIV‐negative adults and children of all ages for a duration of 6 months, conditionally recommended for HIV‐positive adults and children of all ages for a duration of 9 months, and conditionally recommended for all age groups (including both HIV‐negative and HIV‐infected individuals) to be taken daily for 9 months.^152^ The selection of these treatment regimens can be based on patient‐specific factors and the availability of healthcare resources.

#### 4.1.2 Chemotherapeutic strategies for drug‐sensitive TB

For several decades, the WHO has developed and published standard treatment recommendations for TB, which have been widely adopted worldwide. When following these recommendations, approximately 85% of patients can be successfully treated. According to the WHO's comprehensive guidelines on TB module 4, the recommended first‐line treatment regimen for drug‐susceptible TB patients is 2HRZE/4HR.^153^ In the initial phase, treatment involves the administration of isoniazid (H), rifampicin (R), pyrazinamide (Z), and ethambutol (E) for 2 months, followed by a continuation phase of isoniazid and rifampicin for an additional 4 months. It is preferable to provide daily medication throughout the entire treatment course for newly diagnosed active PTB patients whenever feasible. Furthermore, for newly diagnosed PTB patients treated with the rifampicin‐containing regimen, it is not recommended to extend the intensive phase if sputum smear microscopy remains positive after the intensive phase. Nonsevere TB cases in children and adolescents aged 3 months to 16 years (without suspicion or evidence of MDR‐TB or RR‐TB) should receive a 4‐month treatment regimen of 2HRZ(E)/2HR (2 months of isoniazid, rifampicin, and pyrazinamide, with or without ethambutol, followed by 2 months of isoniazid and rifampicin). For individuals coinfected with HIV, regardless of CD4 cell count, antiretroviral therapy should be initiated as soon as possible within 2 weeks after starting TB treatment^153^

#### 4.1.3 Chemotherapy strategies for DR‐TB

The treatment of MDR/RR‐TB patients remains a challenge in TB treatment and exacerbates the global burden of antimicrobial resistance. The increasing incidence of DR‐TB is associated with gaps in detection and prevention, limited care models, and restricted treatment options.^154^ According to the guidelines provided by the WHO in the TB operational handbook, there are several regimens available for the treatment of MDR/RR‐TB patients.^155^

For MDR/RR‐TB patients with confirmed or presumed fluoroquinolone susceptibility, a recommended treatment regimen of 6 months is suggested, which includes bedaquiline (BDQ), pretomanid, linezolid (600 mg), and moxifloxacin (BPaLM).^155^ For MDR/RR‐TB patients without fluoroquinolone resistance, a 9‐month all‐oral regimen is recommended. This 9‐month all‐oral regimen includes BDQ (for 6 months) in combination with levofloxacin/moxifloxacin, ethionamide, ethambutol, high‐dose isoniazid, pyrazinamide, and clofazimine (for 4 months, potentially extended to 6 months if sputum smear remains positive at the end of 4 months), followed by continued treatment with levofloxacin/moxifloxacin, clofazimine, ethambutol, and pyrazinamide (for 5 months).^155^ Linezolid can also be used as a substitute for ethionamide for the initial 2 months of treatment. Additionally, in situations where the BPaLM/BPaL regimen or the 9‐month all‐oral regimen cannot be implemented (e.g., severe extrapulmonary TB, additional resistance to key drugs in the BPaLM/BPaL regimen), consideration should be given to using longer treatment regimens^155^ (Table 3).

**TABLE 3:** Grouping of medicines recommended for use in longer MDR‐TB regimens.

Groups and steps^*^	Medicine and abbreviation	
Group A: Include all three medicines	Levofloxacin or moxifloxacin	Lfx/Mfx
	Bedaquiline	Bdq
	Linezolid	Lzd
Group B: Add one or both medicines	Clofazimine	Cfz
	Cycloserine	Cs
	Terizidone	Trd
Group C: Add to complete the regimen, and when medicines from groups A and B cannot be used	Ethambutol	E
	Delamanid	Dlm
	Pyrazinamidef	Z
	Imipenem–cilastatin	Ipm–Cln
	meropenemg	Mpm

* Group A is the frequently employed treatment regimen for multidrug‐resistant tuberculosis; group B entails the incorporation of one or two drugs to group A; group C regimens may be employed in situations where groups A and B are not suitable.

#### 4.1.4 Novel therapeutic drugs for TB

##### 4.1.4.1 Bedaquiline

BDQ is a diarylquinoline drug and the first new class of anti‐TB medication in over 40 years. Early studies have shown that BDQ exhibits potent selectivity against key ATP synthase enzymes in replicating and dormant MTB, while sparing the activity of ATP synthase in eukaryotic organisms such as humans.^156^, ^157^ Additionally, BDQ has been found to effectively inhibit both drug‐susceptible and drug‐resistant strains of MTB,^158^, ^159^ and it possesses bactericidal activity against dormant (nonreplicating) MTB, whereas isoniazid is inactive against dormant bacteria.^160^, ^161^

In a phase 2b randomized, double‐blind, placebo‐controlled clinical trial for the treatment of MDR‐TB, after 8 weeks of BDQ treatment, the sputum culture conversion rate was 48% (10 out of 21), which was significantly higher than the 9% (two out of 23) in the placebo group.^157^ The results from the second stage of the trial showed that at 120 weeks of treatment, the cure rate was 58% in the BDQ group compared with 32% in the placebo group (*p* = 0.003), with similar rates of adverse events between the two groups.^162^

A phase 3 clinical trial, a single‐arm, open‐label trial, evaluated the efficacy of BDQ in 233 patients with prior treatment failure of MDR‐TB. The trial's primary endpoint was the median time to culture conversion, and the secondary endpoint was the culture conversion rate at 24 weeks of treatment. The results showed a median time to culture conversion of 57 days after BDQ treatment, and 80% of patients achieved culture conversion at 24 weeks of treatment.^162^

##### 4.1.4.2 Delamanid

Delamanid is a drug believed to primarily inhibit the synthesis of mycolic and keto‐mycolic acids, which are important cell wall components in MTB and *Mycobacterium bovis*. Unlike isoniazid, delamanid does not inhibit α‐mycolic acid.^163^, ^164^ As a prodrug, delamanid requires metabolic activation before it can be used in anti‐TB treatment. Studies have shown that delamanid exhibits strong activity against standardized and clinical isolates in vitro, without cross‐resistance or antagonism with drugs such as rifampicin, ethionamide, streptomycin, or isoniazid.^163^, ^165^, ^166^ Research has also found that the bactericidal activity of delamanid is comparable to that of rifampicin.^36^

A study using a mouse model evaluated a 6‐month treatment regimen of delamanid in combination with isoniazid and rifampicin compared with the standard four‐drug therapy (ethambutol, isoniazid, rifampicin, and pyrazinamide).^163^ After 6 months of treatment, no mice in the delamanid group had detectable survival of MTB, while four mice in the standard treatment group still had viable bacteria.^163^ Another randomized controlled trial compared the safety and efficacy of delamanid in combination with the Beijing regimen versus placebo for treating MDR‐TB. The results showed that delamanid increased the sputum culture conversion rate at 2 months for patients with MDR‐TB.^167^ Furthermore, there is an ongoing study funded by Otsuka Pharmaceutical Co., Ltd. evaluating the safety, efficacy, and pharmacokinetics of delamanid in a 6‐month treatment regimen for children with MDR‐TB.^168^

##### 4.1.4.3 Pretomanid

Pretomanid contains a bicyclic nitroimidazole furan, and it is currently undergoing phase III clinical evaluation as part of a drug regimen that includes linezolid and BDQ.^169^, ^170^ In earlier studies, PA‐824, the compound from which pretomanid is derived, has shown efficacy against wild‐type and drug‐resistant strains.^169^ To date, no cross‐resistance with other anti‐TB drugs has been observed.^171^ Additionally, it is effective against nonreplicating bacteria (NRB), making it a viable option for the treatment of LTB.^172^

An exploratory study found that a treatment regimen combining pretomanid with pyrazinamide (Pa) and moxifloxacin (M) (PaMZ) resulted in faster cure rates in mice compared with the standard regimen of isoniazid, pyrazinamide, and rifampicin.^173^ Based on this finding, a prospective randomized proof‐of‐concept study was conducted in previously untreated PTB patients, evaluating several new drug combinations, including a regimen with PaMZ treatment.^174^ The study found that the PaMZ regimen had the most potent early bactericidal activity among the new drug combinations, in striking contrast to the standard regimen (including pyrazinamide, rifampicin, isoniazid, and ethambutol). Currently, the second‐stage trial of bactericidal activity provides the strongest evidence for the PaMZ regimen and regimens containing pretomanid.^52^ The results demonstrate that in patients with drug‐sensitive TB, Pa200MZ (PaMZ with pretomanid at a daily dose of 200 mg) exhibits stronger bactericidal activity compared with conventional treatment. Additionally, patients receiving PaMZ treatment have a higher sputum culture conversion rate at 8 weeks compared with those receiving standard treatment. In patients with MDR‐TB, the Pa200MZ regimen shows bactericidal activity similar to that of drug‐sensitive TB patients receiving standard treatment. These findings suggest that this new treatment regimen holds promise for rapidly and effectively treating MDR‐TB, although further research is still needed.^175^ Médecins Sans Frontières (MSF) conducted a 6‐month evaluation of various combinations of BDQ, pretomanid, linezolid, moxifloxacin, and clofazimine for the treatment of MDR‐TB in a pragmatic study that has received ethical and regulatory approval.^176^ As of now, pretomanid remains a potential novel drug that holds promise for changing the treatment approach to MDR‐TB, particularly as part of new treatment regimens.^175^, ^177^

##### 4.1.4.4 Outlook and future directions

The development of novel anti‐TB drugs is a challenging undertaking. The treatment of TB typically involves the simultaneous use of multiple drugs, making the development of effective drug combination regimens more complex than that of single drugs. Despite some progress in recent years in the development of anti‐TB drugs, numerous challenges persist, rendering the ultimate goal of TB eradication still difficult to achieve. The development of anti‐TB drugs necessitates the continuous exploration of new compounds and mechanisms to address drug resistance and complexity. Moreover, there is a need to enhance clinical trial capabilities globally to evaluate the efficacy of new drugs and compounds rapidly. In addition to intensifying efforts in anti‐TB drug development, there is a need to shift treatment paradigms and explore novel treatment methods and strategies to enhance treatment effectiveness and reduce the transmission of TB. This is of paramount importance for the ultimate eradication of TB.

### 4.2 Immunotherapy for TB

TB is not only an infectious disease but also an immune‐related disease. As a novel potential treatment approach, immune therapy can suppress or even eliminate MTB by modulating the immune system of individuals with LTBI and ATB patients. Immune therapy can eradicate dormant bacteria within immune cells by enhancing the innate immune system, shortening the course of the disease, enhancing the killing effect on MTB, and preventing the occurrence of XDR‐TB.^146^ Various immune therapy methods and vaccine administrations have been attempted and have achieved varying degrees of success. Currently, immune therapy for TB shows promising prospects in developing immune checkpoint inhibitors (ICIs), cytokine therapy, and therapeutic vaccines. The ultimate goal of immune therapy is to assist the host in controlling or eradicating MTB and reducing the course of TB disease.

#### 4.2.1 Cytokine treatment

MTB is an intracellular pathogen primarily residing in monocytes and macrophages.^178^, ^179^ Therefore, cellular immune responses play a crucial role in controlling and combating MTB infection. MTB‐specific T cells are integral components of the immune response against TB. These T cells can produce cytokines and effector molecules that modulate innate and adaptive immune responses by influencing cell development, trafficking, and function. The significance of cellular immunity has been widely recognized in treating and controlling TB.^145^ Enhancing cellular immune responses can strengthen the body's ability to resist MTB, aiding in infection control and reducing disease transmission. Currently, cytokines being investigated in clinical studies mainly include IFN‐γ, IL‐2, and GM‐CSF (Table 4). The application of these cytokines holds promise for improving the therapeutic outcomes of TB.

**TABLE 4:** Cytokine therapy program for tuberculosis.

Name	Phase	Sample size	NCT number	Immune mechanism	Immunotherapeutic effect	References
IL‐2	II/ III	500	NCT03069534	Promote proliferation and transformation of CD4+ T cells and NK cells.	Treatment success and cure rates were better in the rhIL‐2 group than in the control group (69.7 vs. 56.6%, 55.6 vs. 37.2%).	^180^
	IV	1100	NCT04766307	NA	NA	NA
IFN‐γ	I/II	30	NCT00001407	Increasing the relative number of CD8 cells reduces fever and sputum bacterial loads.	NA	NA
	I/II	78	NCT05065905	NA	NA	NA
GM‐CSF	II	NA	NA	Reduced growth of Mtb in human monocyte macrophages.	A trend toward faster turnaround was observed in the rhuGM‐CSF group at week 8 (*p* = 0.07). At week 6, seven of the 14 patients treated with rhuGM‐CSF had negative cultures compared with five of the 14 patients in the placebo group (*p* = 0.44).	^181^

##### 4.2.1.1 IFN‐γ and TNF‐α

IFN‐γ and TNF‐α are cytokines with antiviral, antitumor, and immune‐regulatory properties. They are key Th1‐type cytokines involved in controlling MTB infection. Based on their roles in TB immunity, IFN‐γ and TNF‐α have been used to diagnose and immunize TB.^182^ Studies using gene knockout mouse models have shown that the absence of IFN‐γ and TNF‐α promotes the progression of MTB infection in mice.^183^, ^184^ Furthermore, different administration routes have been shown to influence the therapeutic effects of IFN‐γ and TNF‐α. For example, inhalation of 100 μg of IFN‐γ or TNF‐α via aerosol administration can inhibit and significantly reduce MTB growth in the lungs of mice.^185^ These two cytokines act additively or synergistically during the induction of bacteriostasis, with IFN‐γ also contributing to the initiation of TNF‐α secretion.^186^ In a study by Denis et al.,^185^ the therapeutic potential of IFN‐γ and TNF‐α administered via the aerosol route was tested in MTB‐infected mice, resulting in a significant reduction in pulmonary microbial burden, with infected mice surviving completely after 60 days of infection. It has been shown that aerosol delivery of IFN‐γ in TB‐infected patients during the first 2 months of standard chemotherapy can lead to a reduction in fever and sputum bacterial load after 1 week, and improvement in lung consolidation after 2 months.^187^

However, clinical trial results regarding the use of IFN‐γ in the treatment of MDR‐TB patients have shown only transient effects.^188^ Additionally, in an open‐label randomized trial, IFN‐α administered via aerosol with a dose of 3 million units three times per week during the first 2 months of standard chemotherapy showed reduced fever and sputum bacteriological load after 1 week, and improvement in lung consolidation after 2 months in MTB‐infected patients.^189^ Furthermore, two phase I/II clinical trials (NCT00001407 and NCT05065905) were conducted between 1999 and 2006 to investigate the effects of different doses of IFN‐γ in treating MDR‐TB and the efficacy and safety of IFN‐γ in treating HIV and PTB patients. Both trials have been completed, but their results have not been published yet.

##### 4.2.1.2 IL‐2

IL‐2 plays a promoting role in the proliferation and activation of MTB antigen‐specific T cell clones. It can stimulate T cells to secrete IFN‐γ and activate NK cells and macrophages, thereby enhancing macrophage killing of MTB. As early as 1988, several studies have demonstrated that IL‐2 immunotherapy can significantly inhibit the growth of MTB in mouse models.^190^, ^191^ According to Johnson et al.,^192^ in 1995, low‐dose recombinant human IL‐2 (rhuIL‐2) combined with anti‐TB drugs began to be explored for the treatment of PTB. The study found that in patients with refractory PTB or MDR‐TB, about 60% of patients showed a reduction or clearance of sputum bacillary load, which was associated with enhanced activation of the immune system.^193^ However, data from a double‐blind, placebo‐controlled clinical trial suggested that daily intradermal injections of rhuIL‐2 did not significantly enhance bacterial clearance or improve symptoms in drug‐sensitive TB patients.^194^ Thus, the clinical outcomes of rhuIL‐2 combined with chemotherapy for the treatment of PTB or MDR‐TB are inconsistent.

Meta‐analysis results have shown that rhuIL‐2 immunoadjuvant therapy is safe for PTB/MDR‐TB patients and can promote the proliferation and transformation of CD4^+^ T cells and NK cells, leading to an increased sputum smear conversion rate in PTB/MDR‐TB patients. However, there was no significant improvement in radiological changes.^195^ A clinical trial conducted at Nanjing Medical University in China evaluated the enhanced therapeutic and immune effects of rhIL‐2 combined with standard treatment compared with standard treatment alone for PTB/MDR‐TB (NCT03069534). The study showed that the cure rate and MTB clearance rate in the rhIL‐2 combination therapy group were significantly higher than those in the standard anti‐TB treatment control group. It also improved Th1/Th17 immune responses without safety issues in MDR‐TB patients.^180^ In addition, a clinical trial initiated by Beijing Chest Hospital (NCT04766307) is currently ongoing to compare the efficacy and safety of a standardized treatment regimen of 2HRZE/4HR combined with IL‐2 versus the standardized treatment regimen alone in new diagnostic smear‐positive PTB patients. The primary endpoint of this study is the proportion of sputum culture conversion at the end of treatment. Currently, the trial is recruiting volunteers.

These research findings suggest that IL‐2 immunotherapy may serve as an effective strategy for TB treatment, but further research and clinical trials are needed for support and validation.

##### 4.2.1.3 GM‐CSF

GM‐CSF is a cytokine that has immune‐activating and regulatory effects and is widely secreted by various cells. In an MDR‐TB mouse model, immunotherapy with IL‐2 and GM‐CSF can improve the survival rate of mice, reduce bacterial loads in the lungs, spleen, and lesions, and enhance the efficacy of first‐line anti‐TB drugs.^196^ A phase II clinical trial showed that rhuGM‐CSF as adjunctive immunotherapy had better safety and tolerability in treating APTB patients, and rapid sputum conversion could be achieved at week 8 of treatment.^181^ In addition, recombinant GM‐CSF adenovirus used in gene therapy has significantly reduced pulmonary bacterial load compared with conventional chemotherapy in mouse models.^197^

In addition to IFN‐γ, TNF‐α, IL‐2, and GM‐CSF, other cytokines such as IL‐7, IL‐12, IL‐15, IL‐24, and IL‐32 have shown certain effects in the treatment of TB but are still in the research stage. Cytokine therapy as an adjunctive treatment for TB may help enhance the host's immune response. However, cytokines have a short half‐life, leading to higher treatment costs. It is also important to note that cytokine therapy may cause adverse reactions such as fever, headache, and fatigue, and serious immune‐related complications may occur. Therefore, the use of cytokine therapy needs to carefully consider its risks and benefits.

#### 4.2.2 Immune checkpoint inhibitors

Immune checkpoints are regulatory pathways the immune system employs to suppress self‐reactive immune responses. These pathways function by modulating the immune response of T cells through either inhibitory or stimulatory pathways. Typically, ligand–receptor interactions transmit inhibitory or stimulatory signals to T cells, thereby dampening or enhancing T cell‐mediated immune responses.^198^ In recent years, with the rapid advancement of immunotherapy, ICIs have garnered increasing attention in the context of anti‐TB research.^199^ Notably, immune checkpoint proteins currently studied in clinical research include programmed cell death‐1 (PD‐1), CTLA‐4, lymphocyte activation gene‐3 (LAG‐3), T‐cell immunoglobulin and mucin domain‐3 (TIM‐3), and glucocorticoid‐induced tumor necrosis factor receptor.^200^ These immune checkpoint modulators play a significant role in improving immune responses and enhancing treatment efficacy. However, further research is warranted to elucidate their precise effects and potential mechanisms in TB treatment.

##### 4.2.2.1 PD‐1

PD‐1 is an inhibitory cell surface receptor expressed in T cells, B cells, NK T cells, DCs, and activated monocytes.^201^, ^202^ It has two ligands, PD‐L1 (programmed cell death ligand 1) and PD‐L2. The binding of PD‐1 to PD‐L1 or PD‐L2 can regulate the intensity and duration of immune responses by inhibiting T cell activity.^203^ Inhibition of PD‐1 promotes protective multifunctional T cells (PFTs), bacterial clearance, and disease resolution.^204^ Studies have found that MTB infection can induce high expression of PD‐L1, enabling immune evasion through the PD‐1/PD‐L1 pathway.^205^ The mechanism of action of PD‐1 can be divided into two aspects: on one hand, when PD‐1 binds to TCRs, it inhibits T cell activation.^134^ On the other hand, similar to CTLA‐4, PD‐1/PD‐L1 promotes the differentiation and expansion of Treg cells, thereby suppressing the body's immune response.^136^ Furthermore, PD‐1 inhibitors can enhance the function of T lymphocytes in active PTB, promote cytotoxicity of CD8+ T lymphocytes, increase the release of IFN‐γ and TNF‐α, thereby reducing necrosis of macrophages and controlling MTB infection.^204^, ^205^

##### 4.2.2.2 CTLA‐4

CTLA‐4 is a member of the immunoglobulin superfamily and is expressed on activated T cells along with the costimulatory protein CD28. Both molecules can bind to CD80 and CD86 on the surface of DCs, but CTLA‐4 has a higher affinity and avidity for CD80 and CD86 compared with CD28. While CD28 delivers stimulatory signals, CTLA‐4 outcompetes CD28 in binding to CD80 and CD86, thereby inhibiting T cell function. T cells are the main effector cells in cytotoxicity.^206^, ^207^ When CTLA‐4 binds to its ligands CD80/CD86, it inhibits T cell activity through downstream signaling pathways such as PI3K–AKT and MEK–ERK. Inhibition of these signaling pathways results in cell cycle arrest, reduced cell proliferation and differentiation and suppressed generation of effector factors required for immune responses. Therefore, blocking the binding of CTLA‐4 to its ligands can relieve immune suppression and enhance T cell activity and immune responses.

##### 4.2.2.3 LAG‐3

LAG‐3 is an inhibitory coreceptor with a structure similar to CD4. LAG‐3 can suppress Th1 immune responses by activating Treg cells, promoting their proliferation, and inhibiting monocyte differentiation. Both Treg cells and monocytes exert downstream inhibitory effects on the activation, proliferation, and function of Th1 effector T cells.^208^, ^209^, ^210^ Inhibiting LAG‐3 signaling leads to increased antigen presentation, thereby enhancing Th1 immune responses and increasing the production of IFN‐γ.^211^

Studies have shown that LAG‐3 is significantly upregulated (approximately 100‐fold) in human and macaque lungs during the active PTB stage, and it is specifically localized to groups of T cells, including Treg cells and NK cells. The expression of LAG‐3 in macaque lungs is associated with higher mycobacterial burden.^212^, ^213^ Furthermore, LAG‐3 is highly expressed in granulomas of TB, in macaques with ATB, as well as in animals with LTBI reactivated by simian immunodeficiency virus (SIV) coinfection, while it is not expressed in the lungs of animals with LTBI or infected with SIV or other pulmonary bacterial pathogens other than mycobacteria. These cells also coexpress IL‐10. Existing research suggests that LAG‐3, as a known regulator of Th1 responses, has potential implications in TB.^208^, ^209^ Therefore, there is a compelling rationale to investigate the role of LAG‐3 in regulating immune responses in TB.

##### 4.2.2.4 TIM‐3

TIM‐3 is a type I transmembrane protein that consists of an extracellular membrane‐distal N‐terminal immunoglobulin variable domain, a membrane‐proximal mucin domain, a transmembrane domain, and a cytoplasmic tail.^214^ Initially identified as being associated with the inhibition of IFN‐γ production by CD4^+^ and CD8^+^ T cells, recent research has shown that it also plays an important role in innate immune cells.^215^ Studies have found that TIM‐3 is constitutively expressed on the surface of monocytes in mice and humans, with higher expression on DCs.^216^ On DCs, signaling via high levels of TIM‐3 synergizes with TLR signaling on the cell surface, promoting DC activation and the generation of pro‐inflammatory cytokines and the activation of immune T cells.^216^, ^217^ Additionally, increased expression of TIM‐3 has been observed in humans and nonhuman primates with active PTB.^218^

Studies have shown that during MTB infection in mice, in addition to TIM‐3, other inhibitory receptors such as PD‐1 are coexpressed. Blocking TIM‐3 can restore T cell function and improve bacterial control, particularly in susceptible mice with chronic infection. Furthermore, treatment with anti‐TIM‐3 antibodies in chronically infected mice resulted in reduced bacterial burden and increased production of T cell cytokines, indicative of T cell activation.^218^ TIM‐3 is unlikely to be the sole molecule mediating T cell exhaustion, but targeting TIM‐3 blockade is considered an effective strategy against TB.

ICIs, as an emerging immunotherapy strategy, have demonstrated promising potential in the treatment of TB. However, it is important to note that ICIs may elicit immune‐related adverse events such as immune overactivation or autoimmune reactions.^219^ Furthermore, several research groups have reported cases where ICI therapy resulted in the development of ATB as a side effect.^219^, ^220^ In particular, Lee et al.^221^ first reported this phenomenon.

#### 4.2.3 Vaccine development and strategies

Therapeutic vaccines for TB aim to modulate or selectively induce the immune system of individuals infected with MTB, restoring immune balance, suppressing immune damage, and enhancing immune responses to inhibit or eradicate the pathogen. These vaccines offer advantages such as ease of administration, convenience, cost effectiveness, and minimal side effects. Currently, there are several therapeutic vaccines in clinical development, including MV, MIP, DAR‐901, RUTI, M72/AS01E, H56:IC31, and AEC/BC02 (Table 5).

**TABLE 5:** TB therapeutic vaccine research pipeline.

Name	Phase	Sample size	NCT number	Immune mechanism	Immunotherapeutic effect	References
MV	II	40	NCT01380119	Enhancement of cellular immunity in patients with anti‐TB infection	After 1 month, Mtb clearance was 8% in the MV‐treated group and 5% in the control group.	^222^
	III	1000	NCT01979900		NA	NA
MIP	III	1020	NCT00265226	Activates natural immunity and stimulates T‐cell immune response	After 4 weeks of treatment, the rate of sputum culture conversion was significantly higher in the MIP group (67.1%) than in the placebo group (57%).	^223^
	III	1400	NCT00810849		The incidence rates were 25.0 and 24.3% in the MIP and placebo groups, respectively.	^224^
DAR‐901	I	59	NCT02063555	Enhances Th1 cytokine response and improves immunity	Increase sputum conversion rate and promote lesion resorption	^225^
	II	625	NCT02712424		In the placebo IGRA converter (*p* = 0.03), the DAR ‐ 901 IGRA converter had a median ESAT ‐ 6 response of 50.1 spot‐forming cells (SFCs) and 19.6 SFCs.	^226^
RUTI	I	24	NCT00546273			^227^
	II	95	NCT01136161	Induction of mixed TH1/TH2/TH3, multiantigen reactions without local or systemic toxicity	RUTI elicits multiantigen reactions, especially against 16 and 38 kDa antigens	^228^
	III	9	NCT02711735	NA		NA
M72/AS01E	I	12	NCT00730795		Good safety	^229^
	II	110	NCT00397943	Stimulates strong M72‐specific humoral and CD4+ T‐cell responses		^230^
	II	3575	NCT01755598		HIV‐negative LTBI adults provided 54% protection efficiency	^231^
	II	402	NCT04556981		NA	NA
H56:IC31	I	25	NCT01967134		Has an acceptable safety profile and induces antigen‐specific IgG and CD4+ T cell responses expressing Th1‐type cytokines	^232^
	II	84	NCT02378207	Induction of IgG and CD4 + T cells to express Th1‐type cytokines		^233^
	I/II	51	NCT02503839			^234^
	IIa	98	NCT01865487		H56: IC31 vaccine induces a durable antigen‐specific CD4 T cell response	^235^
	IIb	900	NCT03512249		NA	
ID93/GLA‐SE	IIa	60	NCT02465216	Induction of a strong and long‐lasting Th1‐type immune response	ID93+GLA‐SE induced significantly higher antigen‐specific IgG and CD4 T cell responses than placebo	^236^
	IIa	107	NCT03806686		NA	NA
AEC/BC02	I	25	NCT03026972	Induction of long‐term antigen‐specific cellular immune responses	NA	NA
	I	30	NCT04239313		NA	NA

##### 4.2.3.1 MV vaccine

MV is a therapeutic vaccine derived from inactivated *Mycobacterium vaccae* and is used as an adjunctive treatment for active PTB. Research has shown that the MV vaccine can protect mice models from MTB infection.^237^ Preclinical studies have demonstrated that administration of inactivated MV in mice can elicit Th1‐dominant or mixed Th1/Th2 immune responses while also activating cytotoxic CD8+ T cells with potential protective properties that can kill MTB‐infected macrophages.^237^, ^238^, ^239^ A meta‐analysis revealed that MV can effectively increase sputum conversion rates, but its impact on lesion resolution, cavity closure, and mortality rates has been inconsistent, possibly due to variations in dosing frequency and intervals.^240^ Therefore, it is necessary to determine the most effective dosing regimen and the long‐term effects of MV.

In China, a modified MV vaccine named Vaccae™ has been developed through collaboration between pharmaceutical research institutions and hospitals, utilizing high‐pressure jet cutting technology.^240^ Vaccae™ obtained the Chinese Drug New Certificate in 1999 (Certificate No.: (1999) S‐03) and was approved by the China Food and Drug Administration as a vaccine for adjunctive treatment of TB (Approval No.: S20010003).^8^, ^9^, ^241^ Originally produced by Anhui Zhifei Longcom Biologic Pharmacy Co. Ltd. (now Anhui Zhifei Biopharmaceutical Co. Ltd.), Vaccae™ has played an important role in enhancing immunity, promoting phagocytosis, regulating bidirectional immune responses, and reducing pathological damage. Clinically, it has been used as an adjunctive therapy for TB.^242^, ^243^ A phase II clinical trial conducted in 2013 (NCT01380119) showed that after 1 month of treatment with MV (V7) tablets, acid‐fast bacilli disappeared significantly in the sputum smears of TB patients, though long‐term effects still require further observation.^222^ Currently, a phase III clinical trial (NCT01979900) is being conducted in Guangxi Zhuang Autonomous Region, China, to evaluate the effectiveness and safety of Vaccae™ in preventing TB among individuals with LTBI.

Vaccae™ is currently the only TB immunotherapeutic drug recommended by the WHO, although it may cause local skin rash, induration, or fever as adverse reactions in a very small number of individuals.^241^, ^244^

##### 4.2.3.2 MIP vaccine

The MIP vaccine is a vaccine prepared from nonpathogenic rapidly growing mycobacteria and can activate innate immunity and stimulate T cell immune responses by inducing TLR signaling pathways.^245^, ^246^ In small animal models, it has been demonstrated that the MIP vaccine can reduce organ mycobacterial burden and is associated with early cell‐mediated immune augmentation, including increased cytotoxic T cells and balanced Th1/Th2 immune responses in the later stages of chemotherapy.^247^ Furthermore, MIP has been shown to be safe in retreatment TB patients^223^ and can activate NF‐KB through TLR‐4 signaling, leading to the secretion of pro‐inflammatory cytokines and NO by infected macrophages, thereby promoting protective immune responses.^245^ It has been reported that TB patients receiving directly observed treatment, short‐course combined with the MIP vaccine showed faster sputum clearance compared with patients receiving standard anti‐TB therapy alone.^248^, ^249^

However, in a phase III clinical trial conducted in TB pericarditis patients, two‐thirds of whom had TB‐HIV coinfection, the MIP vaccine did not show significant therapeutic effects but demonstrated significant adverse events. Pus formation at the injection site was observed in 15% of patients, and there was a higher incidence of Kaposi's sarcoma in HIV‐positive patients (NCT00810849).^224^ Another phase III randomized, double‐blind, placebo‐controlled, multicenter clinical trial sponsored by the Department of Science and Technology, India, and Cadila Pharmaceuticals Ltd. evaluated the therapeutic efficacy and safety of MIP in Indian Type II TB patients (NCT00265226).^96^ The study observed that there was no significant difference in sputum smear conversion rate between patients receiving MIP treatment and the placebo group after 2 weeks, with rates of 53.35 and 48.72%, respectively. However, after 4 weeks of treatment, the MIP group showed a significantly higher sputum culture conversion rate (67.1%) compared with the placebo group (57%), indicating the ability of MIP to clear bacteria.^223^

##### 4.2.3.3 DAR‐901 vaccine

DAR‐901 is an inactivated whole‐cell vaccine derived from *M. vaccae* and represents a novel scalable production process for producing SRL172.^100^ This vaccine can induce a Th1 immune response and generate faster and stronger specific immune responses against structural and growth‐related antigens, thereby reducing the MTB burden and decreasing pulmonary pathology.^250^ A phase I clinical trial (NCT02063555) demonstrated that patients developed long‐lasting scars with intradermal administration of DAR‐901, and injection of DAR‐901 combined with chemotherapy for 3−12 times improved sputum smear conversion rates, promoted lesion absorption, and enhanced Th1 cytokine responses.^225^ In April 2016, a randomized, placebo‐controlled, double‐blind phase II clinical trial was conducted among previously BCG‐vaccinated adolescents in the United Republic of Tanzania (NCT02712424). The trial results showed that DAR‐901 had good safety but did not prevent initial or sustained conversion of IGRA.^226^ Participants who received DAR‐901 and experienced IGRA conversion exhibited enhanced immune responses to ESAT‐6.^226^

##### 4.2.3.4 RUTI vaccine

RUTI is a vaccine composed of liposomes cultured under anaerobic and stress conditions, containing MTB‐detoxified fragments known to induce the expression of a wide range of oxidative stress proteins.^251^ Its mechanism of action involves triggering immune responses against these antigens to enhance the therapeutic effect against NRB, which are believed to contribute to treatment duration and relapse.^252^, ^253^ RUTI can induce humoral immune responses and a mixed Th1/Th2/Th3 cellular immune response without local or systemic toxicity.^253^ Animal studies have shown that RUTI alone is ineffective against ATB animal models and may lead to immune damage. However, good results may be obtained when using RUTI to treat infected animals after chemotherapy.^254^

In human studies, phase I/II clinical trials (NCT00546273 and NCT01136161) have been conducted on the RUTI vaccine in combination with chemotherapy for treating LTBI. The trial results showed that the RUTI vaccine was well‐tolerated, and a single dose of 25 mg of RUTI could induce immune responses against multiple antigens, particularly the 16 and 38 kDa antigens, which are biomarkers associated with LTBI, thus effectively inducing cellular immune responses in LTBI volunteers. However, adverse reactions were dose‐dependent, and injection site nodules were commonly observed.^145^, ^228^ Additionally, a phase IIa clinical trial (NCT02711735) aimed to study the safety and immunogenicity of administering RUTI therapeutic vaccine to MDR‐TB patients after successful intensive phase treatment. Unfortunately, this trial has been terminated. In future RUTI clinical trials, a major consideration is improving the vaccine to reduce adverse reactions in volunteers and determine the appropriate vaccine dosage range.

##### 4.2.3.5 M72/AS01E vaccine

M72/AS01E is a subunit TB candidate vaccine developed by GlaxoSmithKline (GSK), consisting of the highly immunogenic MTB proteins Mtb39A and Mtb32A, along with the AS01E adjuvant.^255^ Phase I/IIa clinical trials (NCT00730795 and NCT00397943) have demonstrated that M72/AS01E has good clinical tolerability and induces strong M72‐specific humoral immune responses and CD4^+^ T cell responses but weaker CD8^+^ T cell responses.^230^ A subsequent phase IIb clinical trial (NCT01755598) showed that M72/AS01E provided 54% efficacy in reducing the incidence of TB in HIV‐negative adults with LTBI.^255^ However, during a 36‐month follow‐up, the final efficacy was found to be 49.7%.^231^ Last, after 3 years of follow‐up, it was found that M72/AS01E vaccination provided at least 3 years of immune protection against the development of ATB from LTBI. Currently, a randomized, placebo‐controlled phase III clinical trial (NCT04556981) is underway in South Africa to evaluate the safety and immunogenicity of M72/AS01E in HIV‐positive participants receiving viral suppression and antiretroviral therapy.

##### 4.2.3.6 H56:IC31 vaccine

H56:IC31 is a subunit vaccine composed of three MTB antigens (Ag85B, ESAT‐6, and Rv2660c) and the IC31 adjuvant produced by Valneva Austria GmBH.^232^ Studies have shown that vaccination with H56:IC31 can prevent bacterial reactivation and significantly reduce bacterial burden in both LTBI and ATB mouse or NHP models compared with control groups.^256^ The safety and immunogenicity of H56:IC31 have been evaluated in four clinical trials (NCT01967134, NCT02378207, NCT02503839, and NCT01865487). The results have demonstrated that the vaccine has good safety profiles and can induce antigen‐specific IgG antibodies and CD4^+^ T cell immune responses, producing Th1‐type cytokines. A phase I/IIa clinical trial conducted in South Africa (NCT01865487) evaluated the optimal dose and efficacy of the vaccine in MTB‐infected and uninfected adults. The results showed that two or three doses of the lowest dose (5 μg H56/500 nmol IC31) could induce persistent antigen‐specific CD4^+^ T cell responses in both MTB‐infected and uninfected adults.^235^ In another randomized, open‐label phase I/II clinical trial (NCT02503839), the safety and immunogenicity of H56:IC31 were primarily assessed in patients with pulmonary and extrapulmonary TB, as well as its combination with a cyclooxygenase‐2 (COX‐2) inhibitor (etoricoxib). The results showed that H56:IC31 could induce expansion of antigen‐specific T cells and a higher proportion of antigen‐specific seroconversion.^234^ Although H56:IC31 and etoricoxib demonstrated similar safety and immunogenicity, the coadministration with the COX‐2 inhibitor weakened the immune response, suggesting a potential antagonistic effect of the two as host‐directed therapy.^234^ Currently, a phase IIb double‐blind, randomized, placebo‐controlled clinical trial (NCT03512249) is ongoing to evaluate further the safety and efficacy of H56:IC31 in reducing TB recurrence in HIV‐negative adults. The trial aims to recruit 900 participants but has not yet recruited volunteers.

##### 4.2.3.7 ID93+GLA‐SE vaccine

ID93+GLA‐SE vaccine is developed by the Infectious Disease Research Institute (IDRI) in the United States. It consists of three MTB virulence‐associated antigens (Rv2608, Rv3619, Rv3620), one latency‐associated antigen (Rv1813), and the GLA‐SE adjuvant.^257^ When combined with chemotherapy, this vaccine has been shown to induce strong and long‐lasting Th1 cell immune responses, prolong survival, reduce bacterial burden in organs, decrease pathological damage, and enhance the efficacy of chemotherapy in mouse and monkey TB models.^258^, ^259^ A stable inhalable powder formulation of ID93/GLA‐SE has also been developed as an alternative to injection administration.^260^

Furthermore, a phase IIa randomized, double‐blind, placebo‐controlled clinical trial (NCT02465216) conducted in Cape Town, South Africa, evaluated the safety and immunogenicity of the ID93+GLA‐SE vaccine in HIV‐negative adult TB patients after completion of treatment. The results showed that the ID93+GLA‐SE vaccine induced strong and persistent antibody responses and antigen‐specific multifunctional CD4^+^ T cell responses. Among the vaccine groups that received two doses of 2 μg ID93 + 5 μg GLA‐SE, antigen‐specific IgG and CD4^+^ T cell responses were significantly higher compared with the placebo group, and these immune responses persisted for 6 months. No vaccine‐related serious adverse events were observed.^236^ Currently, a phase IIa randomized, double‐blind, placebo‐controlled clinical trial (NCT03806686) is ongoing in South Korea to evaluate the safety, immunogenicity, and efficacy of the ID93+GLA‐SE vaccine in healthy healthcare workers who have received BCG vaccination. The study has not yet recruited volunteers.

##### 4.2.3.8 AEC/BC02 vaccine

AEC/BC02 is a recombinant subunit TB vaccine that consists of two main components: MTB Ag85b and a fusion protein called ESAT6‐CFP10. It is combined with a CpG and aluminum‐based adjuvant system referred to as BC02.^261^ Preliminary evaluations of the immunogenicity and efficacy of the AEC/BC02 vaccine in a BALB/c mouse model have demonstrated its ability to induce strong cellular immune responses, leading to high frequencies of specific IFN‐γ‐secreting T cells in mice.^261^ Subsequently, a study validated the therapeutic effect of the AEC/BC02 vaccine in LTBI mice, showing that immunotherapy with AEC/BC02 significantly reduced bacterial burden in the lungs and spleens of mice, possibly attributed to the specific IFN‐γ and IL‐2 cell‐mediated immune responses induced by AEC/BC02.^262^ A phase I clinical trial (NCT03026972) was conducted in 2017 to evaluate the safety of the AEC/BC02 vaccine. Additionally, a phase Ib, single‐center, single‐dose, placebo‐controlled clinical trial (NCT04239313) was conducted in Hubei, China in January 2020 to assess the safety and immunogenicity of the AEC/BC02 vaccine in healthy adults. In contrast to NCT03026972, NCT04239313 enrolled subjects who tested negative for TB‐PPD and IGRA, and preliminary evaluations of the safety of low‐dose vaccine and adjuvant were performed. Both of these clinical trials have completed participant recruitment, but the results have not yet been released.

Developing therapeutic vaccines for TB is an important global public health endeavor. While several candidate vaccines have entered the clinical trial phase, a widely accepted and ideal therapeutic vaccine for TB has yet to emerge. The development of effective TB vaccines still faces numerous technological and scientific challenges. Nevertheless, research and efforts in this field continue to progress in the pursuit of safer and more effective therapeutic vaccines for TB. This remains a global priority to alleviate the burden of TB and improve global health conditions.

## 5 CHALLENGES AND PROSPECTS

**FIGURE 3:**
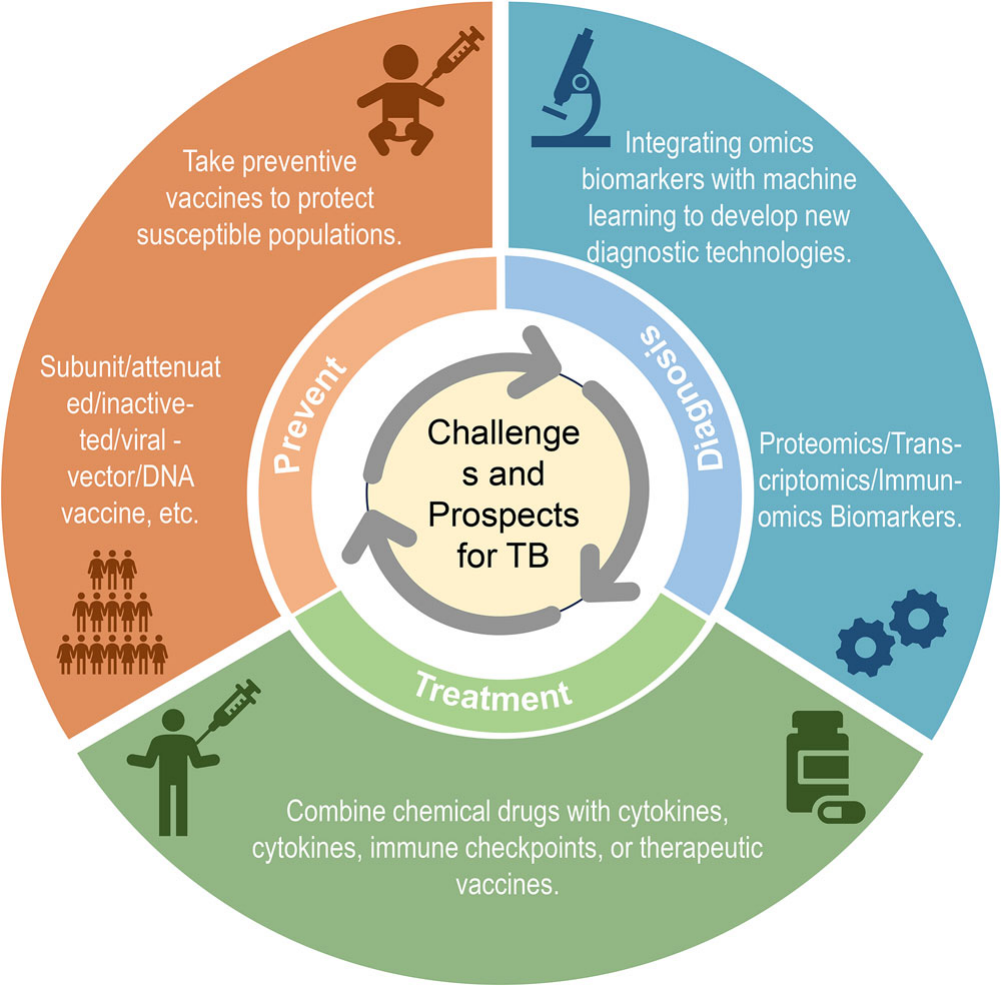
Challenges and prospects for TB control. Currently, controlling the source of infection, interrupting transmission pathways, and protecting susceptible populations are key measures in tackling MTB infection. However, preventative, diagnostic, and therapeutic interventions for TB are also important in reducing the burden of the disease. First, in terms of preventing MTB infection, the BCG vaccine, the only known vac‐cine with some protective effects, has limited efficacy and duration of protection in adults. Therefore, the development of multiple vaccines with preventative efficacy may offer new strategies for susceptible populations. Second, in the diagnosis of MTB infection, current diagnostic technologies lack high specificity and sensitivity and cannot differentiate LTBI from ATB. Therefore, the identification of biomarkers related to immune mechanisms for diagnostic purposes may be an important goal in improving tuberculosis diagnosis efficiency. Last, in the treatment of MTB infection, challenges arise from the effectiveness of conventional chemotherapy drugs and the emergence of MDR‐TB. Thus, the use of immunomodulatory cytokines, immune checkpoint inhibitors, and therapeutic vaccines in combination with anti‐TB chemotherapy drugs may represent effective approaches for TB treatment. MTB, *Mycobacterium tuberculosis*; TB, tuberculosis; BCG, Bacillus Calmette‐Guérin; LTBI, latent tuberculosis infection; ATB, active tuberculosis; MDR‐TB, multdrug‐resistant tuberculosis.

TB is a significant infectious disease that poses a threat to global population health and remains the leading cause of death from a single infectious agent worldwide.^263^ The immune system plays a crucial role in human defense against MTB invasion, a relationship that has been recognized for centuries. With the advancement of the global economy and scientific knowledge, immune‐based approaches to combat TB have garnered public attention and made some progress. However, these approaches also face certain challenges. Nonetheless, these challenges provide new insights for future efforts in TB control. In the final section of this review, we will delve into the specific challenges and opportunities in the prevention, diagnosis, and treatment of TB (Figure 3).

### 5.1 Prevention of TB

It is well known that the three measures for preventing infectious diseases are controlling the source of infection, interrupting transmission pathways, and protecting susceptible populations. However, due to the long history of MTB and the difficulty in distinguishing between LTBI and ATB, protecting susceptible populations becomes the most challenging aspect of these measures. As mentioned earlier, the only vaccine currently used for TB prevention is the BCG vaccine, which has limited efficacy and duration of protection in adults (approximately 10 years).^264^, ^265^ A study found that administering preventive TB vaccines can significantly reduce the incidence of ATB disease. Vaccination after effective exposure can prevent around 30−40% of TB cases, while vaccination before exposure can prevent approximately 20% of TB cases.^266^ Therefore, the lack of preventative TB vaccines presents both challenges and opportunities for research on novel vaccines.

In the development of novel TB vaccines, preventative TB vaccines can be classified into five categories: subunit vaccines, attenuated vaccines, inactivated vaccines, viral vector vaccines, and DNA vaccines. First, subunit TB vaccines mainly consist of immunologically active components from MTB and are often used as booster doses after initial BCG vaccination to enhance the protective effect or prolong the effective titers provided by BCG. Although subunit vaccines have the advantages of high efficiency, safety, and low cost, they require the addition of immunostimulants (adjuvants) to induce immune protection or immunotherapy, thus ultimately exerting their preventive role in TB.^267^ Second, attenuated TB vaccines are prepared by removing certain virulence genes from MTB. These avirulent strains of MTB can significantly express various antigens, activating different T cell types and triggering complex and diverse immune responses to achieve long‐term protection.^268^ However, attenuated TB vaccines may carry potential risks of regaining virulence and complications related to immune complexes. Finally, viral vector TB vaccines introduce protective antigens from MTB into the body using relatively safe viral vectors, achieving effective and sustained immune protection.^269^ Viral vector TB vaccines not only have high safety, ease of production, and low cost but also can carry larger gene fragments. However, viral vector TB vaccines also have drawbacks, such as the potential for regaining virulence and unstable expression of exogenous genes. Additionally, TB DNA vaccines are an innovative type of vaccine that provides immune protection against MTB by eliciting immune responses in the host.^178^, ^179^, ^180^ TB DNA vaccines typically induce specific humoral and cellular immune responses, thus playing a role in preventing or treating TB.^270^

In summary, various novel vaccines have different effects in preventing MTB infection. However, the limitations of these vaccines cannot be ignored, which is also the reason why no licensed vaccines are currently available. Therefore, after scientists tackle these challenges and conduct clinical trials, these preventative TB vaccines may become important interventions in combating MTB infection.

### 5.2 Diagnosis of TB

Currently, methods for diagnosing MTB infection in clinical practice mainly include sputum smear microscopy, sputum culture, imaging techniques, the TST, and IGRA, among others. However, as detailed in the previous text, these methods have limitations that pose significant challenges to the occurrence and development of TB. Therefore, this review explores the potential of protein, genetic, and immune biomarkers as new opportunities for the clinical diagnosis of TB.

First, proteomics is an emerging omics technology primarily used to identify the expression and interactions of different proteins in cells, and it is widely employed in the diagnosis, prediction, and treatment of TB.^271^ Proteomics can reflect the actual production of proteins in cells, providing new approaches and means to differentiate ATB from LTBI. However, when using proteomics for diagnosing TB, the use of multiple antigens as models needs to be considered, which may increase the economic burden on patients.

Second, transcriptomics is an important component of genetic biomarkers. It provides information by analyzing the transcriptional differences in immune cells and other cells during MTB infection. Transcriptomics can reflect the host's transcriptional levels during different stages of infection, including microarray and RNA‐seq technologies. However, although transcriptomics can accurately detect specific targets, it cannot analyze certain new biomarkers.

Last, immune biomarkers, mainly represented by cytokines, of which IFN‐γ is widely recognized, have led to the IGRA based on T‐cell immune response becoming the most commonly used test for TB diagnosis. Immune biomarkers show promising applications in TB diagnosis, such as using combinations of various cytokines to differentiate different MTB infection statuses. However, immune biomarkers also have limitations in distinguishing LTBI from ATB during clinical diagnosis.

Although the emergence and research of the aforementioned biomarkers have brought revolutionary changes to TB diagnosis, it should be noted that the sensitivity and specificity of these biomarkers may vary among different populations.^272^ Therefore, in the face of these issues, machine learning algorithms such as random forests, support vector machines, decision tree classification, single‐layer perceptrons, and multilayer perceptrons can be applied for validation to improve the sensitivity and specificity of diagnosis, laying the foundation for future research.

### 5.3 Treatment of TB

In recent years, researchers worldwide focused on new strategies for the prevention and diagnosis of TB and the challenges faced in TB treatment. Early TB treatment primarily emphasized the use of combination chemotherapy, following principles of early initiation, regularity, completeness, dosage adequacy, and combination therapy, divided into the intensive and consolidation phases. In the previous text, we elaborated on the first‐line treatment regimen for drug‐susceptible TB, yet its effectiveness is limited in cases of MDR‐TB. Consequently, researchers have shown interest in studying cytokines, immune checkpoints, and therapeutic vaccines.

First, during host defense against MTB infection, T cells produce cytokines and effector molecules to eliminate the bacteria. Among these cytokines, IFN‐γ and TNF‐α are key Th1‐type cytokines that control MTB infection and play an important role in its clearance.^182^ Additionally, IL‐2 and IL‐24 stimulate the secretion of IFN‐γ by TB antigen‐specific T cells, while GM‐CSF and IL‐32 contribute to the killing of MTB by macrophages. These cytokines play a vital role in eliminating the bacteria, thus attracting considerable attention in TB treatment.^145^

Second, ICIs were originally developed for the treatment of non‐small‐cell lung cancer but are effective against PD‐1 elevation induced by MTB infection.^199^ As a result, ICIs are being explored for TB treatment. Immune checkpoints such as PD‐1, CTLA‐4, LAG‐3, and TIM‐3 are inhibitory proteins that suppress T cell activation upon binding to TCRs, allowing MTB to develop LTBI or ATB. The therapeutic mechanism of ICIs involves acting as a “brake” in the binding between immune checkpoints and TCRs, thereby enhancing T cell activity and immune response. However, this “brake” effect can lead to further developing LTBI into ATB,^273^ providing a new direction for in‐depth research on immunotherapy for TB.

Last, TB treatment can also be achieved through therapeutic vaccine administration. As mentioned earlier, vaccine administration can prevent the progression of MTB infection to ATB, while therapeutic vaccines can serve as adjunctive therapy or prevention of post‐cure relapse.^274^ The main objective of therapeutic vaccines is to modulate or selectively induce the immune system of TB patients, restoring immune balance, inhibiting immune damage, enhancing immunity, and suppressing or killing MTB. Examples of therapeutic vaccines include MV, MIP, DAR‐901, RUTI, M72/AS01E, H56:IC31, and AEC/BC02 (as mentioned earlier). Despite the advantages of simplicity, convenience, affordability, and minimal side effects, these vaccines are still in the early stages of development and have not yet entered full clinical trials. Therefore, further evaluation of the efficacy of these vaccines is needed.

The host immune system plays a crucial role in controlling MTB infection, and immunotherapy has made certain breakthroughs. However, clinical treatment approaches for TB have not undergone significant changes, indicating the presence of obstacles in translating basic research to clinical applications. Therefore, developing new immunotherapeutic drugs and methods, along with their combination with anti‐TB drugs, could potentially represent effective treatment strategies for TB or MDR‐TB.

## 6 CONCLUSIONS

TB is one of the infectious diseases of concern to the WHO, and the emergence of drug‐resistant strains presents significant challenges to TB prevention and control. This review provides an immunological perspective on the immune response and immune evasion characteristics of MTB infection, aiming to enhance our understanding of the immune mechanisms underlying the occurrence and development of TB, as well as to identify effective strategies for prevention, diagnosis, and treatment. With the increasing number of individuals infected with MTB, there is a growing need for diagnostic methods to distinguish between LTBI and ATB. Although three new TST methods (C‐TB, Diaskintest, and EC skin test) and seven new IGRAs (such as AdvanSure™ TB‐IGRA ELISA, Wantai TB‐IGRA, Standard E TB‐Feron, QIAreach QFT, ichroma™ IGRA‐TB, VIDAS TB‐IGRA, and T‐Track TB) have shown excellent performance in diagnosing ATB, they are unable to differentiate between ATB and LTBI. Therefore, biomarkers are receiving attention for TB diagnosis. Currently, biomarkers such as proteomics, transcriptomics, and cytokines are being used to distinguish between LTBI and ATB, but they require the integration of machine learning algorithms to maximize diagnostic accuracy. Thus, future TB diagnostic approaches may focus on the application of biomarkers combined with machine learning.

In terms of TB treatment, vaccination is a cost‐effective and efficient method. However, the protective efficacy of the only available therapeutic TB vaccine, BCG, is limited, highlighting the urgent need for developing novel TB vaccines. Although some progress has been made with therapeutic TB vaccines mentioned in this review, such as MV, MIP, DAR‐901, RUTI, M72/AS01E, H56:IC31, and AEC/BC02, challenges remain, including poor sustainability, difficulty in selecting antigen epitopes, and the exclusion of pregnant women from existing TB vaccine trials. Despite the many challenges in TB vaccine development, we must recognize that developing therapeutic TB vaccines is a public health effort to promote human well‐being. Furthermore, while early combination chemotherapy has limited effectiveness in MDR‐TB, it remains effective for drug‐sensitive TB. Therefore, a combination of research on cytokines, ICIs, therapeutic vaccines, and the application of anti‐TB drugs may provide new avenues and approaches for TB treatment.

In conclusion, despite their challenges, these new research findings may provide further impetus toward achieving the WHO goal of ending global TB by 2035.

## AUTHOR CONTRIBUTION

Li Zhuang, Ling Yang, Linsheng Li, and Zhaoyang Ye worked together to perform the literature search; Li Zhuang, Ling Yang, and Linsheng Li worked together to write the original draft; Wenping Gong and Ling Yang worked together to prepare the figures; Ling Yang, Li Zhang, and Linsheng Li worked together to prepare the tables; all authors discussed the concepts of the manuscript; Wenping Gong performed the supervision and revision. All authors read and agreed to the published version of the manuscript.

## CONFLICT OF INTEREST STATEMENT

The authors declare no conflict of interest.

## ETHICS STATEMENT

Ethics statement was waived because it was a review.

## Data Availability

All data generated or analyzed during this work are included in this published review.
